# The Rac inhibitor HV-107 as a potential therapeutic for metastatic breast cancer

**DOI:** 10.1186/s10020-023-00678-7

**Published:** 2023-06-14

**Authors:** Grace Velez Crespo, Jescelica Ortiz, Eliud Hernández O’Farrill, Cornelis P. Vlaar, Mikhail Inyushin, Yuriy Kucheryavykh, Lilia Kucheryavykh

**Affiliations:** 1 Department of Biochemistry, Universidad Central del Caribe, Bayamón, Puerto Rico; 2 Department of Pharmaceutical Science, School of Pharmacy, University of Puerto Rico, Medical Sciences Campus, San Juan, Puerto Rico; 3 Department of Biochemistry, School of Medicine, University of Puerto Rico, Medical Sciences Campus, San Juan, Puerto Rico; 4 Department of Physiology, Universidad Central del Caribe, Bayamón, Puerto Rico

**Keywords:** Triple-negative breast cancer, Rac GTPase, Proliferation, Metastasis, HV-107

## Abstract

**Background:**

The significant challenge in treating triple-negative breast cancer (TNBC) lies in its high rate of distant metastasis. To address this, inhibiting metastasis formation in TNBC is vital. Rac is a key player in cancer metastasis. Previously, we developed Ehop-016, a Rac inhibitor that successfully reduced tumor growth and metastasis in mice. In this study, we assessed the effectiveness of HV-107, a derivative of Ehop-016, in inhibiting TNBC metastasis at lower doses.

**Methods:**

Rho GTPases activity assays were performed with the use of GST-PAK beads and Rac, Rho, and Cdc42 GLISA. Cell viability was assessed through trypan blue exclusion and MTT assays. Cell cycle analysis was conducted using flow cytometry. To evaluate invading capabilities, transwell assays and invadopodia formation assays were performed. Metastasis formation studies were conducted using a breast cancer xenograft mouse model.

**Results:**

HV-107 inhibited Rac activity by 50% in MDA-MB-231 and MDA-MB-468 cells at concentrations of 250–2000 nM, leading to a 90% decrease in invasion and invadopodia activity. Concentrations of 500 nM and above caused dose-dependent reductions in cell viability, resulting in up to 20% cell death after 72 h. Concentrations exceeding 1000 nM upregulated PAK1, PAK2, FAK, Pyk2, Cdc42, and Rho signallings, while Pyk2 was downregulated at 100–500 nM. Through in vitro experiments, optimal concentrations of HV-107 ranging from 250 to 500 nM were identified, effectively inhibiting Rac activity and invasion while minimizing off-target effects. In a breast cancer xenograft model, administration of 5 mg/kg HV-107 (administered intraperitoneally, 5 days a week) reduced Rac activity by 20% in tumors and decreased metastasis by 50% in the lungs and liver. No observed toxicity was noted at the tested doses.

**Conclusion:**

The findings indicate that HV-107 exhibits promising potential as a therapeutic medication utilizing Rac inhibition mechanisms to address metastasis formation in TNBC.

**Supplementary Information:**

The online version contains supplementary material available at 10.1186/s10020-023-00678-7.

## Background

Breast cancer is the most common cancer worldwide and the second leading cause of cancer death in the United States, with 1 in 8 women at risk of developing it during their lifetime (Sung et al. 2020; Organization 2022; U.S. 2022). Among breast cancer types, triple-negative breast cancer (TNBC) is one of the most aggressive and commonly affects women under 40 (Yin et al. 2020; Markman 2022). Due to the complex heterogeneity of TNBC, targeted therapies are limited (Marmé and Schneeweiss 2015; McArthur 2022). Despite initial responses to chemotherapy, resistance frequently and rapidly occurs, and metastatic tumors arise (Soares et al. 2021; Health 2018). TNBC has the highest rate of distant metastasis among all breast cancer subtypes and is associated with the shortest overall survival, with metastatic breast cancer lacking effective targeted treatment. (Yin et al. 2020; Guo et al. 2020; Metastatic-Breast-Cancer 2022). Metastatic breast cancer still lacks effective targeted treatment, owning the major impact on the morbidity and mortality of patients (Metastatic-Breast-Cancer 2022; Guan 2015; Chaffer and Weingberg 2011).

Metastasis is a complex multistep process that involves invasion in local tissues, intravasation into blood and lymphatic vessels, extravasation within distant organs, transformation to micro-metastasis, forming small cancer nodules, and finally an invasion within the distant tissues, transforming micro-metastases into macro-metastases (Bid et al. 2013). The Rho/Rac (Ras-related C3 botulinum toxin substrate) GTPases are critical to multiple steps in the metastatic cascade and are widely implicated in cancer progression (Wertheimer et al. 2012; Marei and Malliri 2017). Rac is involved in the regulation of cytoskeleton rearrangements, migration, proliferation, survival, differentiation, cell cycle progression, vesicle trafficking and malignant transformation (Wertheimer et al. 2012; Marei and Malliri 2017).

Rac GTPases are promising targets for antimetastatic cancer therapy, as they are a key molecular switch, activated by a variety of stimuli, including growth factors (such as EGF, PDGF, and HGF) and G-protein-coupled receptor ligands (such as SDF- 1*α*, sphingosine-1-phosphate, and bombesin) giving rise to the activation of signalling pathways involved in cell proliferation and invasion (Bos et al. 2007). Rac GTPases (Rac1, Rac2, and Rac3) have been recognized as important nodes in signalling networks, that control malignant transformation and the metastatic dissemination of cancer cells (Casado-Medrano et al. 2018). Rac1 was reported to be overexpressed or hyperactivated in breast cancer, as well as in other various cancers, including prostate, testicular, ovarian, lung and gastric cancers (Bos et al. 2007; Casado-Medrano et al. 2018). In TNBC, Rac overexpression has been shown to regulate the directional movement of tumor cells and to promote stemness, leading to cancer metastasis and recurrence (Liu et al. 2019). In addition, high expression of Rac1 has been associated with epithelial-to-mesenchymal transition (EMT) markers and correlates with poor patient prognosis. Suppression of Ras-induced apoptosis and activation of p21-activated protein kinase1 (PAK1), AKT (protein kinase B), ERK (Extracellular signal-regulated kinase) and NF*κ*B (nuclear factor kappa B) signalling network has been reported as an effect of Rac activity, leading to cancer progression and metastatic dispersal (Mack et al. 2011). Accumulating evidence indicate, that the up-regulation of the Rac effector PAK is implicated in actin polymerization and cell invasion in TNBC (Best et al. 2022). Rac also has been shown to be required for progression from G_1_ to the S phase of interphase and to be involved in the regulation of the G_2_/M phase of the cell cycle (Mack et al. 2011; Humphries-Bickley et al. 2017). In addition, in vivo studies using animal models of lung cancer, myelogenous leukemia and breast cancer demonstrated that deletion/inhibition of Rac resulted in significant reduction in tumor formation (Mack et al. 2011; Castillo-Pichardo et al. 2014). The involvement of Rac in the development of therapy resistance was demonstrated as well (Kazanietz and Caloca 2017; Sun et al. 2021; Goel et al. 2016), as constitutively active Rac1 promotes resistance to anti-estrogens (ER) in therapeutic approaches for estrogen receptor–positive (ERþ) breast cancer patients, and has been associated with the acquired resistance to trastuzumab, used for ErbB2/HER2-positive breast cancer patients (Sun et al. 2021). Additionally, resistance to anti-VEGF (Vascular endothelial growth factor)/VEGFR (Vascular endothelial growth factor receptor) therapy has been assigned to the Rac-GEF (guanine nucleotide exchange factors) P-Rex1(PIP3 Dependent Rac Exchange Factor) (Goel et al. 2016). Anomalous expression and/or activation of GEFs may affect patient outcome prediction and therapeutic management (Lane et al. 2008). Therefore, inhibition of the interaction of Rac with its direct activator GEFs is a viable strategy for metastatic cancer.

Our group previously developed Ehop-016, a small molecule inhibitor of Rac with an IC_50_ of 1* µM* in metastatic breast cancer cells (Dharmawardhanane et al. 2013; Castillo-Pichardo et al. 2014; Montalvo-Ortiz et al. 2012). Building on this work, we tested several Ehop-016 derivatives and identified HV-107 (previously known as compound 11b) as the most viable candidate (Vlaar et al. 2018). The compound showed 2–4 times greater efficiency in inhibiting Rac compared to the parent compound and demonstrated potential as anti-TNBC metastasis therapeutics. The present study aimed to further characterize HV-107 and its potential as novel treatment for TNBC metastasis.

## Methods

Synthesis of HV-107 was previously described (Vlaar et al. 2018; Hernández et al. 2022). The molecular docking of HV-107 and Rac protein was earlier published by our group (Vlaar et al. 2018).

### Cell culture

TNBC cells MDA-MB-231(#HTB-26**)** and MDA-MB-468(#HTB-132) and non-cancerous epithelial mammary cells MCF10A (#CRL-10317) were obtained from American Type Cell Culture Collection (ATCC, Manassas, VA) and used until passage 16. MDA-MB-231 and MDA-MB-468 cells were cultured in DMEM (#D7777, Sigma-Aldrich, St. Louis, MO, USA) supplemented with 10% FBS (# 35-010-CV, Corning, Inc, AZ, USA), pH7.5, at 5% CO_2_ and 37ºC. MCF10A cells were maintained in Ham’s F-12 media (#D8900 Sigma-Aldrich, St. Louis, MO, USA) supplemented with 10% Horse serum (# H1138Sigma-Aldrich, St. Louis, MO, USA), 100 ng/mL cholera toxin B (#C9903, Sigma Aldrich, St. Louis, MO, USA)), 10 ug/mL insulin (#I9278 Sigma-Aldrich, St. Louis, MO, USA) and 20 ng/mL EGF (# 62253-63-8 Sigma-Aldrich St. Louis, MO, USA).

### Rac activity assays

MDA-MB-468 and MDA-MB-231 cells were treated with vehicle (0.1% DMSO), 100, 250, 500, 1000 and 2000 nM HV-107 for 24 h. Active Rac1.GTP pulldowns were performed for cell lysates with PAK1 PBD Agarose beads (#STA-411, Cell Biolabs Inc., San Diego, CA, USA), and protein samples were subjected to western blot analysis with Rac1 antibody (#2465, Cell Signalling, Denver, MA, USA) and analyzed by Image Studio software version 5.2(LI-COR Biotechnology, Lincoln, NE, USA). Analysis of Rac activity on tumors was assessed with Rac Glisa(#BK128, Cytoskeleton, Denver, CO, USA) colorimetric assay as per manufacturer instructions and absorbance read at 490 nm with SpectraMax iD3 (Molecular devices, CA, USA).

### Rho and Cdc42 Glisa activation colorimetric assay

MDA-MB-468 and MDA-MB-231 cells were treated with vehicle (0.1% DMSO), 100, 250, 500, 1000, and 2000 nM HV-107 for 24 h. Lysates were subjected to active Cdc42 (#BK127, Cytoskeleton, Denver, CO, USA) or active RhoA (#BK124, Cytoskeleton, Denver, CO, USA) Glisa colorimetric assay as per manufacturer instructions and absorbance read at 490 nm with SpectraMax iD3(Molecular devices, CA, USA). Total lysates were subjected to western blot analysis and probed with Cdc42 and Rho antibodies (#2466, #2117, Cell Signalling, Denver, MA, USA).

### Analysis of Rac inhibitors mechanism of action

Lysates of MDA-MB-231 cells were incubated for 1 h at 4 °C with pre-treated RacG15A agarose beads (STA-432, Cell Biolabs Inc., San Diego, CA, USA) with 250 nM of HV-107. Protein samples were subjected to western blot analysis with Vav2 antibody (#2848, Cell Signalling, Denver, MA, USA) and analyzed with Image Studio software version 5.2(LI-COR Biotechnology, Lincoln, NE, USA).

### Western blot analysis

Cells were lysed in 1X PathScan Sandwich ELISA lysis buffer (#7018, Cell Signalling, Denver, MA, USA) supplemented with a 1 mM protease inhibitor cocktail. Clarified cell lysates (20 µg), separated by 10% SDS‒PAGE, were transferred to nitrocellulose membranes and probed with FAK, pFAK Tyr 925, PAK1, PAK2, pPAK1Thr 423/pPAK2 Thr 402, BCL2, Cyclin D1 (#3285, #3284, #2602, #2615, #2601, #3498, #2978 Cell Signalling, Denver, MA, USA), Pyk2 (sc-39318, Santa Cruz Biotechnology, Dallas, TX), and pPyk2 Tyr 402 (Santa Cruz Biotechnology, Dallas, TX) antibodies. Imaging and analysis were performed using the Odyssey DLx Imaging System (LI-COR Biotechnology, Lincoln, NE, USA) and Image Studio software version 5.2.

### Cell viability assays

Trypan blue and MTT assays were used for cell viability analysis.3.5 × 10^5^ cells were incubated in indicated concentrations of HV-107 for 72 h, dissociated, and stained with trypan blue (# T8154, Sigma-Aldrich, St. Louis, MO, USA). The percentage of live and dead cells was quantified. For MTT (3-(4,5-Dimethylthiazol-2-yl)-2,5-diphenyltetrazolium bromide)) colorimetric assay 15,000 cells/well were placed in a 96-well plate in triplicates and incubated with indicated concentrations of HV-107 for 72 h. After the incubation period, 10 uL of MTT (stock 5 mg/mL) was added to each well and incubated for 4 h at 37 °C. Formazan crystals were solubilized with 100 uL of DMSO and incubated for 2 h at 37 °C. After incubation plates were read at an absorbance of 570 nm in the SpectraMax iD3(Molecular Devices, CA, USA).

### Cell cycle analysis

Cells were treated with indicated concentrations of HV-107 for 72 h, fixed with 70% ethanol, and incubated with Guava Cell cycle reagent (#4500-0220, Luminex Corp, Austin, TX, USA) for 30 min. After incubation samples were analyzed using the Guava easy Cyte flow cytometer (Luminex, Austin, TX, USA) and Guava InCyte software (GuavaSoft 3.3 Luminex Corporation, Austin, TX, USA).

### Migration and invasion assays

Migration and invasion assays were performed using fluoroblok inserts 8 µM pore size (#351152, Corning, Inc, AZ). Serum-starved cells were placed on the upper insert membrane, supplemented with HV-107 at indicated concentrations. DMEM supplemented with 10% FBS was used at the lower compartment. After 18–24 h (the optimum migration time was determined for each cell line) or 30–36 h (the optimum invasion time was determined for each cell line), cells were fixed with 70% ethanol and stained with propidium iodide (30 ug/mL, #25535-16-4, Sigma-Aldrich, St. Louis, MO, USA). For invasion assays fluoroblocks were coated with a 1:8 diluted Matrigel matrix (#354248 Corning, Glendale, AZ, USA) as per manufacturer instructions. Images were taken using an Olympus CKX53 microscope with a 40 × objective (Olympus, Center Valley, PA). Image J NIH version 1.52 counting tool was used.

### Invadopodia activity assays

Cells were plated on fluorescein-conjugated gelatine-coated glass coverslips (0.4 mg/mL gelatine, #G13187, Invitrogen, Waltham, MA) in medium supplemented with indicated concentrations of HV-107. The optimum incubation time was determined for each cell culture. Then cells were fixed with 4% paraformaldehyde (# 15710, Electron Microscopy Sciences, Hatfield, PA, USA), permeabilized with 3% BSA 1 × TBST with 0.1% Triton-X100 (# 9036-19-5, Sigma-Aldrich, St. Louis, MO, USA,) and stained with phalloidin–tetramethyl–rhodamine (diluted 1:500, #P1951 Sigma-Aldrich St. Louis, MO, USA) and DAPI (diluted 1:5000, #D9542, Sigma Aldrich, St. Louis, MO, USA). Images were visualized using an Olympus Fluoview FV1000 confocal microscope (Olympus Corporation, Center Valley, PA) and analyzed using Image J software version 1.52. For the quantification of invadopodia formation (IF), cells forming invadopodia in each digital image were counted and normalized to the total number of nuclei in the same image. To quantify invadopodia activity (IA), the fraction area of gelatine degradation was measured and the percentage of matrix degraded was normalized to the number of nuclei in each image.

### Tumor establishment

All animal studies were conducted under protocol #042-2018-30-01-IBC-PHA, approved by the UCC Institutional Animal Care and Use Committee, in accordance with the NIH Guideline for the Care and Use of Laboratory Animals. Female athymic nu/nu mice, 4 to 5wk old (Charles River Laboratories Inc, Wilmington, MA) were maintained under pathogen-free conditions in HEPA-filtered cages.

Mice were inoculated subcutaneously at the third mammary fat pad with 5 × 10^5^ GFP-MDA-MB-231 cells in 100 uL of DMEM + Hepes: Geltrex (#354234, BD Biosciences, San Jose, CA) under isoflurane (#NDC 66794–017-25, Bethlehem, PA) inhalation (1–3% in oxygen using an inhalation chamber at 2L/min) to produce orthotopic primary tumors, as described (Humphries-Bickley et al. 2017). After tumor establishment (4 weeks post-inoculation), animals were randomly divided into treatment groups (vehicle [n = 15], and 5 mg/kg HV-107[n = 13]).

### Administration of HV-107

Mice were treated with vehicle (12.5% ethanol, 12.5% Cremophor 12.5%, and 75% PBS, pH 7.4) or 5 mg/kg body weight (BW)HV-107 by intraperitoneal (i.p.) injection in a 100 µL volume, 5 times a week. Treatments continued until sacrifice at day 63 after the beginning of treatment, as previously described (Castillo-Pichardo et al. 2014; Montalvo-Ortiz et al. 2012).

### Whole body fluorescence image analysis and caliper measurement of tumors

Mammary tumor growth was quantified as changes in the integrated density of GFP(Green Fluorescent Protein) fluorescence and caliper measurement. For fluorescence measurement mice were imaged on day 1 of treatment and then once a week thereafter for 9 weeks, using the iBox Explorer 2610 In Vivo Fluorescent Imaging System (iBOX® Explorer UVP, LLC). Tumor fluorescence intensities were analyzed using Image J NIH software version 1.52n, as the pixel intensity (integrated density) was determined from the digital tracing of the fluorescent tumor area at each imaging session. Tumor growth was calculated as the integrated density of fluorescence of each tumor on each day of imaging, relative to the integrated density of fluorescence of the same tumor on day 1 of treatment. Caliper measurements of tumors were performed on day 1 of treatment and then every two weeks thereafter for 9 weeks. Tumor volume (V) was calculated as V = (Length x width^2^)/2.

### Analysis of metastases

Following sacrifice, primary tumors, lungs and livers were excised and analyzed using an Olympus MV10 fluorescence microscope (Olympus Corporation, Center Valley, PA) and Olympus DP71 digital camera as described (Dharmawardhanane et al. 2013). Lungs and livers were imaged on both sides for the identification of metastatic foci formation. Relative metastasis formation was calculated as the integrated density of fluorescence of treated mice relative to the vehicle. The relative number of metastatic foci formed per organ was analyzed. Measurements were performed using Image J (NIH software version 1.52n).

### Toxicity assays

Blood samples were collected from sacrificed mice via cardiac punctured and processed for the analysis of aspartate aminotransferase (AST), alkaline phosphatase (ALP), and alanine transaminase (ALT) activities, used as a direct measurement of toxicity. Liver enzyme analysis was performed using a commercial source (Core-Plus Labs, Puerto Rico). At the end of the study tumors and livers were excised and weighed.

### Statistical analysis

Data analysis was performed through Graph-Pad Prism version 9.2. Differences between groups were analyzed using Student’s t*-*test for comparison between the two groups. One-way ANOVA was used for comparisons between 3 or more experimental groups and two-way ANOVA for comparisons between 3 or more experimental groups under different conditions. Statistical significance (considered statistically significant at p ≤ 0.05) was determined by Dunnetts’s multiple comparisons test and Unpaired t-test with Welch’s correction. Data were expressed as the mean ± SEM.

## Results

### HV-107 inhibits Rac activity in breast cancer cells

**Fig. 1:**
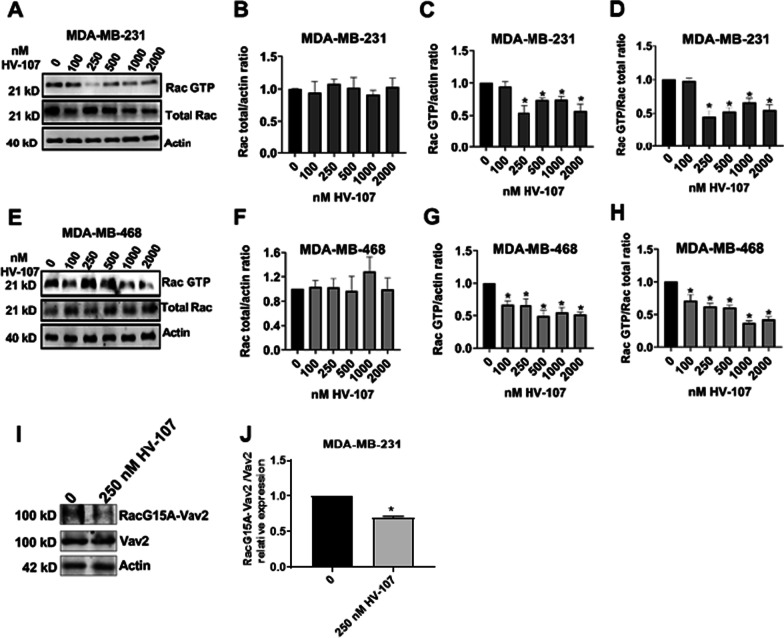
HV-107 inhibits Rac activity in breast cancer cell lines. To examine the effects of HV-107 on MDA-MB-231 (**A**–**D**) and MDA-MB-468 (**E**–**H**) triple negative breast cancer cells, a 24-h treatment with HV-107 ranging from 0 to 2000 nM was conducted. Pulldown assays utilizing the p21-binding domain of PAK were employed to isolate the active form of Rac (GTP bound), followed by western blot analysis. The western blot images presented in panels **A** and **E** illustrate the levels of both total Rac and GTP-bound Rac in MDA-MB-231 and MDA-MB-468 cells treated with various concentrations of HV-107. The quantification of total Rac levels is depicted in panels **B** and **F**, while the quantification of GTP-bound Rac levels is shown in panels **C** and **G**. Panels **D** and H represent the ratio of GTP-bound Rac to total Rac levels. **I**, **J** Pulldowns with RacG15A beads followed by western blots were performed to investigate Rac-Vav2 binding capacity in response to 250 nM HV-107. The western blot images and quantifications depict the levels of Vav2 bound to the RacG15A beads. Actin was used as a loading control. N = 3–4. Error bars represent ± SEM; * p ≤ 0.05

To investigate the potential of HV-107 to inhibit Rac activity in breast cancer cells, MDA-MB-231 and MDA-MB-468 cell lines were chosen due to their different levels of Rac expression and activation: MDA-MB-231 presents an overexpression of Rac, while MDA-MB-468 is characterized with abundant expression of Rac1b (Melzer et al. 2019). In both investigated cell lines, a down-regulation in Rac activity was observed. In MDA-MB-231 cells, a 50% reduction in Rac activity was observed at 250 nM HV-107. In MDA-MB-468 cells, a 40% reduction was observed at 100 nM, and the reduction reached up to 60% at 500 nM. (Fig. 1). There was no observed effect on the total expression of Rac in either of the investigated cell lines. Whole western blot membranes and loading controls are presented in Additional file 1: Fig. S1.

As the parental compound EHop-016 has previously been shown to inhibit Rac activity by disrupting the Rac-Vav2 interaction, the impact of HV-107 on the binding capacity between Rac and Vav2 was investigated using RacG15A pulldown assays combined with western blot analysis of Vav2. Our study revealed that HV-107 reduced the binding of Rac and Vav2 (see Fig. 1I, J). Whole western blot membranes and loading controls are presented in Additional file 2: Fig. S2.

### HV-107 reduces breast cancer cell viability

**Fig. 2:**
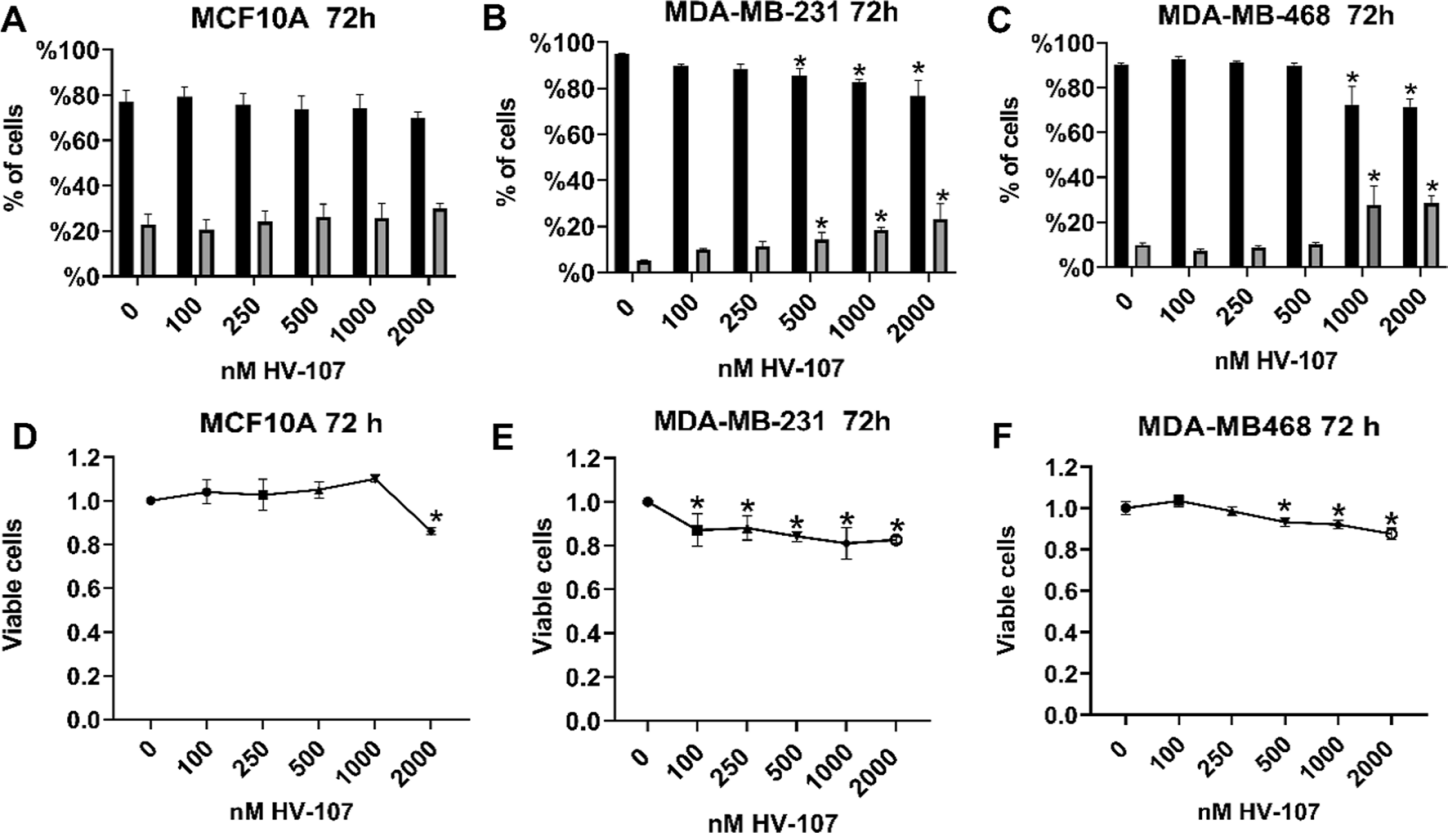
HV-107 affects cell viability in breast cancer cell lines. The results of the trypan blue exclusion assays (**A**–**C**) and MTT assays (**D**–**F**) evaluating cell viability in MCF10A, MDA-MB-231, and MDA-MB-468 cells treated with HV-107 for 72 h are displayed. The relative cell viability is depicted as a percentage of the control (cells treated with the vehicle only). N = 3. Error bars represent ± SEM; significant differences from control (*) are shown (p < 0.05)

The impact of HV-107 on the viability of TNBC cells MDA-MB-231 and MDA-MB-468 cells, as well as non-cancerous mammary epithelial cells MCF10A was evaluated. The cells were subjected to various concentrations of HV-107 for a duration of 72 h, and the viability was measured using both trypan blue exclusion assays and MTT assays. In non-cancerous MCF10A cells (Fig. 2A, D), the use of HV-107 did not affect cell viability in the trypan blue exclusion assays. However, when measured using the MTT assay, a 20% decrease in viable cells.was observed at 2000 nM HV-107. This finding suggests a reduction in cell proliferation without affecting cell death.

Both assays demonstrated a gradual decrease in the viability of MDA-MB-231 cells ranging from 15 to 25% across a concentration range of 100–2000 nM HV-107. Similarly, in MDA-MB-468 cells, a decrease in viability was observed across a range of 500–2000 nM HV-107. These findings indicate that the effect on the viability of TNBC cells is likely due to a combination of increased cell death and reduced proliferation.

### HV-107 does not affect cell cycle progression in breast cancer cells

**Fig. 3:**
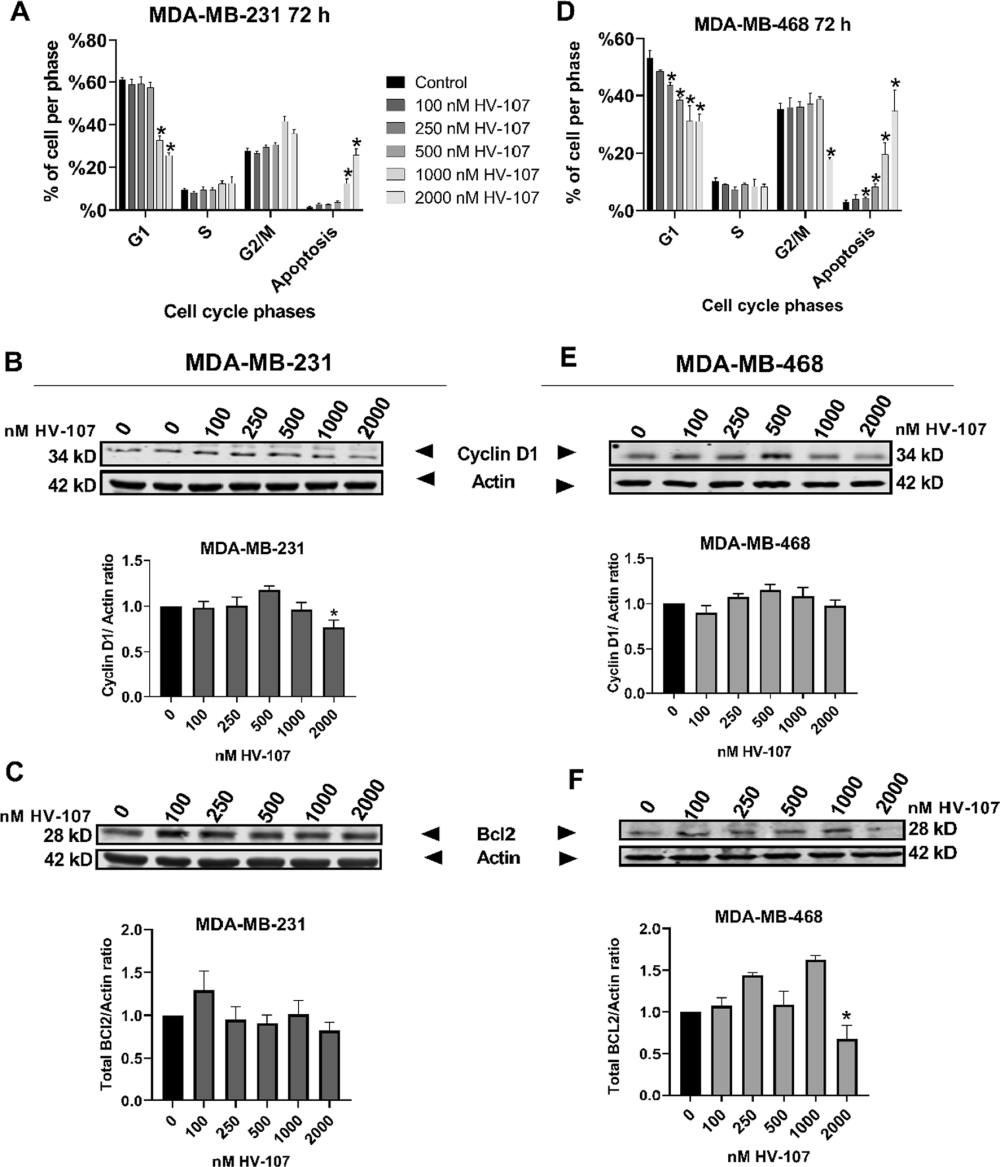
HV-107 does not significantly affect cell cycle progression. Flow cytometric analysis using propidium iodide as a nuclear marker was conducted to assess cell cycle progression in MDA-MB-231 (**A**) and MDA-MB-468 (**D**) cells. The DNA content was used to determine the percentage of cells in the G0/G1, S, and G2/M phases. Bar graphs represent the overall distribution of cells across different phases of the cell cycle. The relative expression of cyclin D1 and Bcl2 in MDA-MB-231 (**B**, **C**) and MDA-MB-468 (**E**, **F**) cells was examined using western blot analysis. The results were analyzed by comparing the fold change in expression relative to the control condition. N = 4. Means and error bars represent ± SEM, and significant differences from control (*) are shown (p < 0.05)

The flow cytometric analysis of DNA content provided insights into the effect of HV-107 and HV-118 on cell proliferation and apoptosis. Cells were subjected to HV-107 treatment for 72 h at the concentrations identified above. In both MDA-MB-231 and MDA-MB-468 cells, HV-107 treatment led to a decrease in the number of cells in the G1 phase, while the cell numbers in the S and G2-M phases remained largely unaffected. Furthermore, an accumulation of cells in the sub-G1 phase, which is indicative of apoptosis, was noticed. These findings suggest that HV-107 does not significantly impact cell cycle progression but instead triggers apoptosis in cells at the G1 stage. The activation of apoptosis was observed at doses exceeding 1000 nM in MDA-MB-231 cells and doses exceeding 250 nM in MDA-MB-468 cells (Fig. 3A, D).

To additionally validate the observed effects on the cell cycle and apoptosis, the expression levels of cyclin D1 and B-cell lymphoma2 (Bcl2) proteins were assessed by western blot. The results revealed that HV-107 treatment generally did not have an effect on cyclin D1 expression in the investigated cell lines, except at the highest concentration of 2000 nM. At this concentration, there was a 20% decrease in cyclin D1 expression observed in MDA-MB-231 cells but not in MDA-MB-468 cells (Fig. 3B, E). This observation aligns with the cell cycle analysis data, suggesting that HV-107 primarily does not influence cell cycle progression within the concentration range of 100–1000 nM. Importantly, these concentrations were previously identified as not affecting the viability of non-cancerous MCF10A cells (Fig. 2). Likewise, the expression of Bcl2 (Fig. 3C, F) was predominantly unaffected by HV-107 treatment in both investigated cell lines, except at the 2000 nM concentration. At this concentration, a reduction in Bcl2 expression was observed in MDA-MB-468 cells but not in MDA-MB-231 cells. These findings indicate that the apoptosis triggered by HV-107 can be initiated through pathways and mechanisms that do not necessarily rely on Bcl2 activity. Complete western blot images and loading controls are presented in Additional file 3: Fig. S3.

### HV-107 reduces migration and invasion in breast cancer cells

**Fig. 4:**
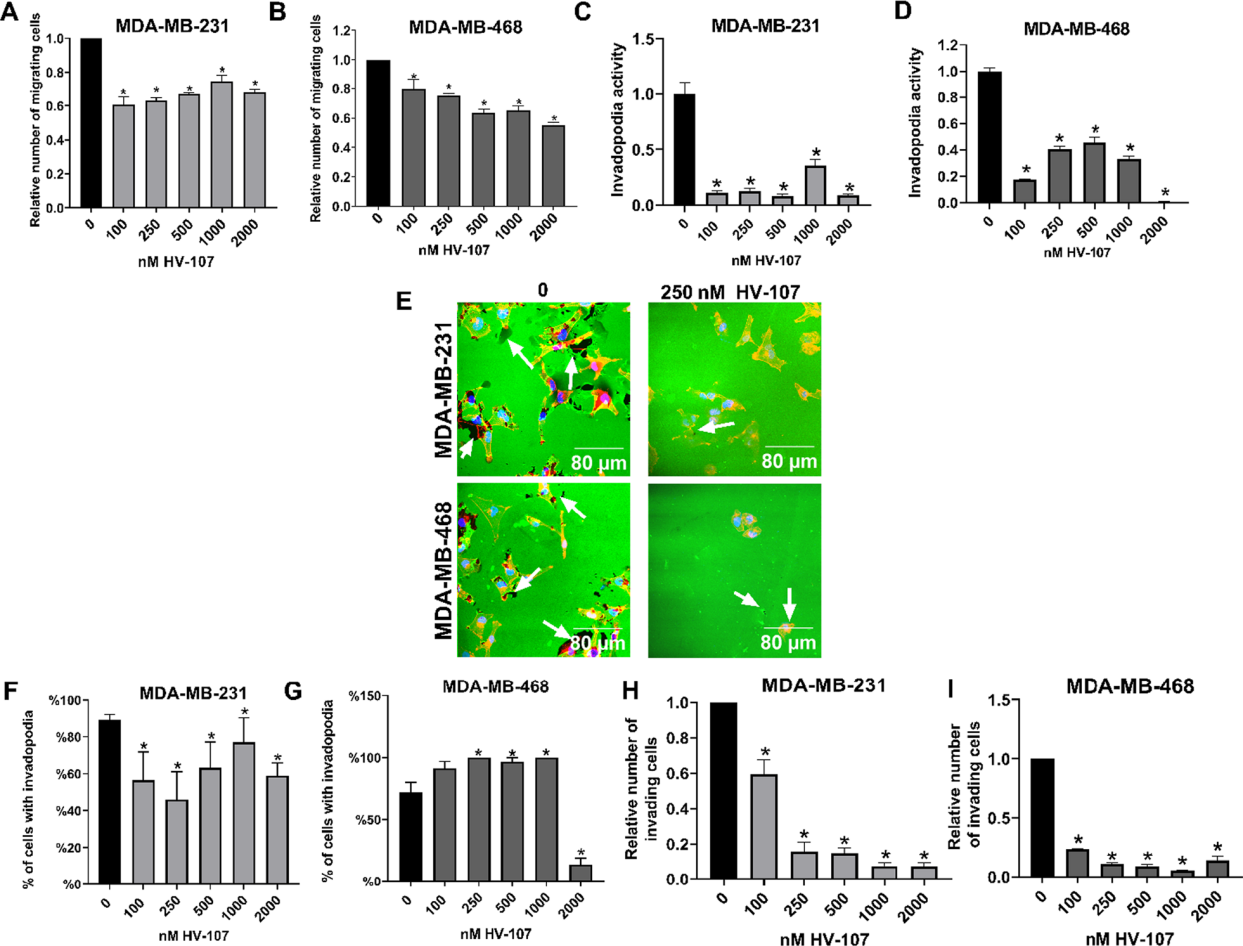
HV-107 treatment downregulate migration, invasion and invadopodia activity in breast cancer cells. Transwell migration and invasion assays were performed for MDA-MB-231 (**A**, **H**) and MDA-MB-468 (**B**, **I**) cells, treated with HV-107 at concentrations ranging from 0 to 2000 nM for 18–24 h respectively. In the invasion assays, transwells, coated with Matrigel, were employed, whereas in the migration assays matrigel was not utilized. The relative number of migrated and invaded cells is presented. Invadopodia formation assays are presented as confocal images (**E**). The area fraction of gelatin matrix (indicating invadopodia activity) was calculated and presented (**C**, **D**) together with calculations of the number of cells forming invadopodia (**F**, **G**), for cells treated with HV-107. F-actin, stained with rhodamine–phalloidin (red), FITC-conjugated gelatin (green), and DAPI, used for nuclei staining (blue), are shown. The degraded areas of FITC-labeled gelatin are depicted as black patches marked by white arrows. Oil immersion objectives (40 ×) were used. Scale bar: 80 µm. Mean ± S.E. and significant differences from control (*) are shown (p < 0.05). N = 5

To evaluate the impact of HV-107 on the motility of TNBC cells, transwell migration assays were conducted for both MDA-MB-231 and MDA-MB-468 cells. The optimal migration times were determined to be 18 h for MDA-MB-231 cells and 24 h for MDA-MB-468 cells. These time points were selected to capture the most effective assessment of cell motility in each cell line. Figure 4A illustrates a significant reduction in migration, approximately 30–40% compared to the control, in MDA-MB-231 cells treated with HV-107 at concentrations of 100 nM and higher. The highest inhibition of migration was observed within the range of 100–250 nM.

Similarly, Fig. 4B demonstrates a 20–40% reduction in migration in MDA-MB-468 cells treated with HV-107 at concentrations of 100 nM and above. The highest inhibition of migration occurred at concentrations ranging from 500 to 2000 nM (40%). These findings indicate that the optimal concentration range for inhibiting migration in both MDA-MB-231 and MDA-MB-468 cells is between 100–500 nM of HV-107. (Representative images are presented in Additional file 4: Fig.S4).

To assess the impact of HV-107 on the ability of cells to degrade the extracellular matrix, invadopodia formation assays were conducted. The number of cells formed invadopodia (IF) and the percentage area of gelatin matrix degradation (IA) were evaluated. The optimal assay durations were determined to be 26 h for MDA-MB-231 cells and 30 h for MDA-MB-468 cells. These time points were identified as the most suitable for capturing and analyzing invadopodia formation and gelatin matrix degradation in each respective cell line. Treatment with HV-107 at concentrations of 100 nM and above resulted in a significant reduction, up to 95%, in the IA compared to untreated cells (Fig. 4C, E, F). In addition, in MDA-MB-231 cells, there was a 50% reduction in the number of cells forming invadopodia (IF) upon HV-107 treatment. In contrast, MDA-MB-468 cells treated with HV-107 exhibited an increase in the number of cells forming invadopodia (Fig. 4G), while also demonstrating a strong reduction in the percentage area of gelatin matrix degradation (IA) (Fig. 4D, E, H). Complete images are provided in Additional file 5: Fig. S5. To assess the effects of HV-107 on the complex process of invasion, which involves both cell migration and extracellular matrix degradation, invasion assays were conducted (Fig. 4H, I). Consistent with the results obtained from the cell migration assays and IA analysis, the invasion assays demonstrated a gradual reduction in the number of invading cells treated with HV-107. In MDA-MB-231 cells, the decrease ranged from 40 to 90%, while in MDA-MB-468 cells, the reduction ranged from 80 to 90%. These effects were observed at HV-107 concentrations of 100 nM and above. The results indicate that HV-107 effectively inhibits the invasive properties of both MDA-MB-231 and MDA-MB-468 cells. Representative images are presented in Additional file 5: Fig. S5. The observed effects of HV-107 on cell migration and extracellular matrix degradation are consistent with the inhibition of Rac activity, as shown in Fig. 1. It is noteworthy that even at the lowest concentrations of HV-107, resulting in minimal inhibition of Rac activity, a significant reduction in invadopodia activity was observed.

These finding highlights that even a modest 30% inhibition of Rac activity, observed at HV-107 concentrations of 100–250 nM, can exert a substantial influence on the remodeling of the extracellular matrix and the invasive capacity of breast cancer cells.

### Effects of HV-107 on Rac-related signaling circuit and cytoskeletal rearrangement

**Fig. 5:**
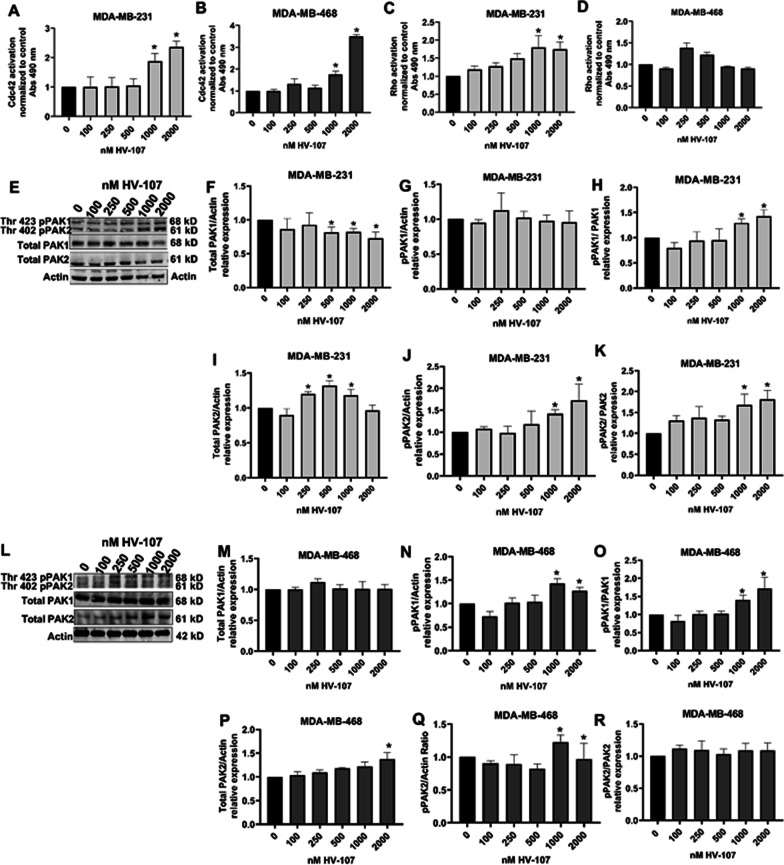
HV-107 at 1000 nM impact Rac-related GTPases and PAK1/2 effectors on breast cancer cells. Triple negative breast cancer cells MDA-MB-231 and MDA-MB-468 were subjected to HV-107 treatment at various concentrations (ranging from 0 to 2000 nM) for a duration of 24 h. GTP-bound Cdc42 (**A**, **B**) and GTP-bound Rho (**C**, **D**) were assessed using the Cdc42 GLISA and Rho GLISA assays, respectively. In panels **E** and **L**, western blot images depict the expression of total and phosphorylated PAK1 and PAK2 in MDA-MB-231 and MDA-MB-468 cells treated with HV-107. The quantification of total PAK1 and PAK2 (**F**, **I**, **M**, and **P**) and phosphorylated PAK1 (Thr 423) and PAK2 (Thr 402) (**G**, **J**, **N**, and **Q**) are shown. The ratios of phosphorylated PAK1 and PAK2 to total PAK levels are displayed in panels **H**, **K**, **O**, and **R**. N = 4. Error bars represent ± SEM; * p ≤ 0.05

To evaluate the effect of HV-107 on Rac-related signaling pathways, GLISA and western blot analyses were performed. These analyses aimed to measure the levels of total and GTP-bound Cell division control protein 42 (Cdc42) and Rho GTPases. Additionally, the levels of total and phosphorylated forms of PAK1 and PAK2, Focal adhesion kinase (FAK), and Proline-rich tyrosine kinase 2 (Pyk2) were examined. In both cell lines, an elevation in Cdc42 activity was observed when treated with HV-107 at concentrations of 1000 nM and above (Fig. 5A, B). Furthermore, HV-107 in the range of 1000–2000 nM resulted in an increase in Rho activity specifically in MDA-MB-231 cells, whereas no significant effect on Rho activity was detected in MDA-MB-468 cells (Fig. 5C, D). No substantial impact on the total expression levels of Cdc42 and Rho was observed in either cell line (Additional file 6: Fig. S6; Additional file 7: Fig. S7). The enhanced activation of Cdc42 and Rho, triggered by HV-107 treatment, coincided with noticeable changes in cellular morphology. In both cell lines, treatment with HV-107 at concentrations of 1000 nM and above led to an increase in filopodia length. Additionally, in MDA-MB-231 cells, there was an augmented formation of stress fibers. These alterations in the cellular structure were consistent with the up-regulation of Rho and Cdc42 activity. Additional file 8: Fig. S8 showcases confocal images depicting the presence of stress fibers and filopodia formation in both cell lines, further supporting the association with the increased activation of Rho and Cdc42.

PAK activity was evaluated following the administration of HV-107, considering that PAK acts as a downstream effector shared by Rac and Cdc42 (Bid et al. 2013). In MDA-MB-231 cells, a notable reduction of 20% in total PAK1 protein levels was observed alongside an accompanying increase in phosphorylated pPAK1 (Thr 423) levels, specifically at HV-107 concentrations of 500 nM and above. Consequently, the overall expression of pPAK1 (Thr 423) remained relatively unchanged. Conversely, in MDA-MB-468 cells treated with HV-107, no significant alterations in total and phosphorylated PAK1 were observed, except for the highest concentrations of 1000–2000 nM (Fig. 5E–R).

A modest increase in total PAK2 expression was detected in MDA-MB-231 cells treated with HV-107 at concentrations ranging from 250 to 1000 nM. In contrast, no significant changes in total PAK2 expression were observed in MDA-MB-468 cells at HV-107 concentrations below 2000 nM (Fig. 5). Moreover, no significant differences in pPAK2 (Thr 402) expression were observed upon HV-107 treatment compared to the control group, at concentrations below 1000 nM, in both cell lines. The results suggest that HV-107 affects the regulation of PAK1 and PAK2 primarily at concentrations exceeding 1000 nM. The accompanying loading controls for the western blot analysis can be found in Additional file 9: Fig. S9, Additional file 10: Fig. S10 and Additional file 11: Fig. S11.

**Fig. 6:**
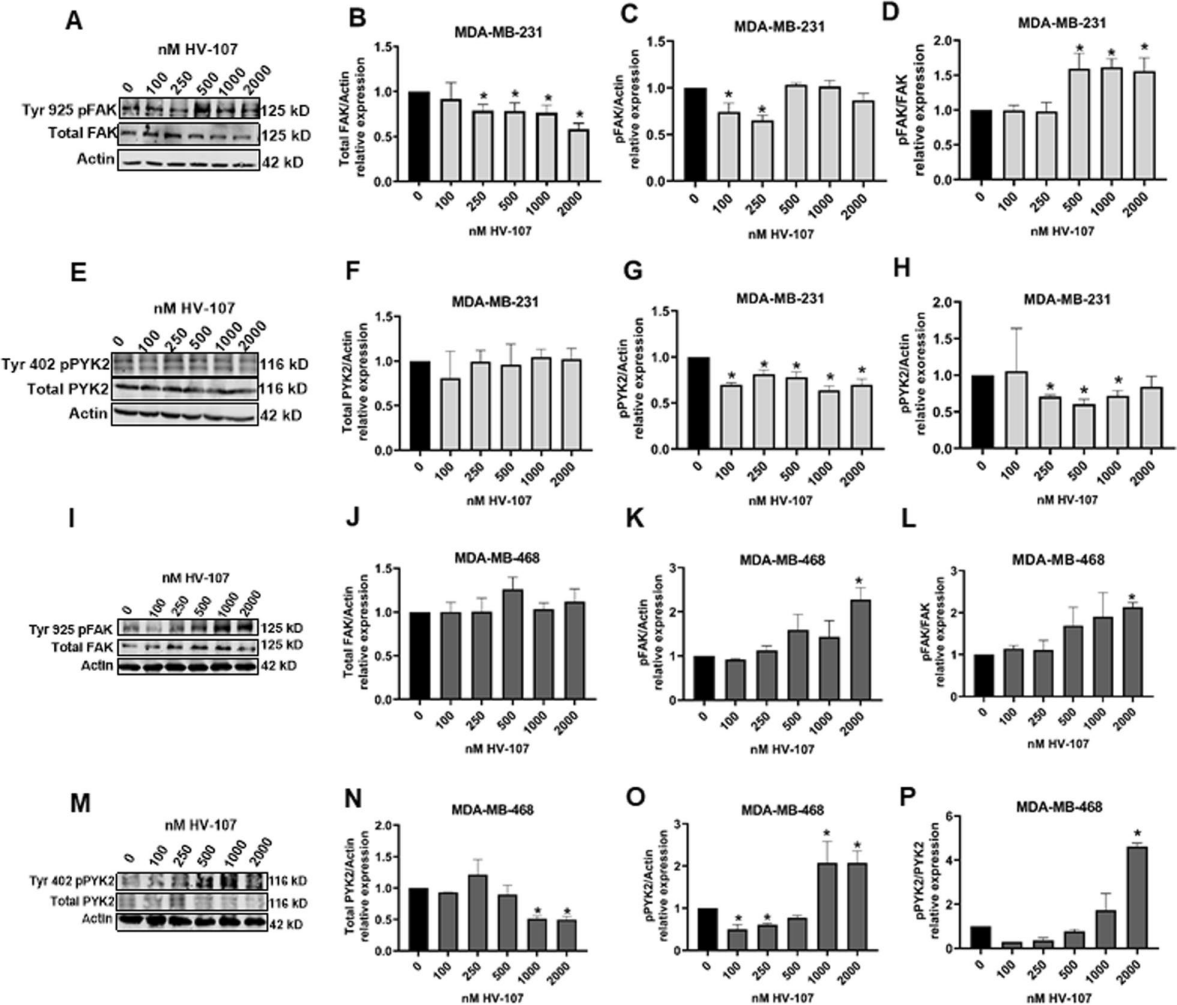
HV-107 affects Rho GTPases, FAK and Pyk2 signaling in breast cancer cells. Triple negative breast cancer cells MDA-MB-231 (**A**–**H**) and MDA-MB-468 (**I**–**P**) were exposed to HV-107 at concentrations ranging from 0 to 2000 nM for a duration of 24 h. The western blot images (**A**, **E**, **I**, **M**) and quantifications for the expression of total and phosphorylated FAK and Pyk2 (**B**–**P**) are shown. N = 3. Error bars represent ± SEM; * p ≤ 0.05

The impact of HV-107 on the activation of FAK and its homolog Pyk2 was investigated, considering their potential role in modulating Rac and Rho activity by activating Rho-GAPs and GEFs (Mack et al. 2011; Subik et al. 2010). In MDA-MB-231 cells, the total expression of FAK protein decreased in a dose-dependent manner upon HV-107 treatment, accompanied by an upregulation of phosphorylation at Tyr 925 at concentrations of 500 nM and above. This led to no significant changes in the total levels of phosphorylated FAK in this cell line. In contrast, no notable alterations in total and phosphorylated FAK were observed in MDA-MB-468 cells at concentrations below 2000 nM (Fig. 6).

The results indicated that HV-107 treatment had no significant effect on the total expression of Pyk2 in both MDA-MB-231 and MDA-MB-468 cells at concentrations below 1000 nM (Fig. 6). However, a 30% reduction in Pyk2 phosphorylation at Tyr402 was observed in MDA-MB-231 cells within the range of 100–2000 nM HV-107. In MDA-MB-468 cells, there was up to a 50% reduction in Pyk2 phosphorylation at concentrations below 500 nM, followed by a 100% up-regulation of Pyk2 phosphorylation at concentrations above 500 nM (Fig. 6). The complete western blot membranes with corresponding loading controls can be found in Additional file 12: Fig. S12, Additional file 13: Fig. S13 and Additional file 14: Fig. S14. The findings indicate that HV-107 may have an impact on FAK and Pyk2 and their phosphorylation.

The effects on FAK signalling are predominantly observed at concentrations above 1000 nM, while the effects on Pyk2 signalling are observed across the entire range of tested concentrations.

### HV-107 reduces tumor growth and metastasis formation in a xenograft mouse model of breast cancer.

**Fig. 7:**
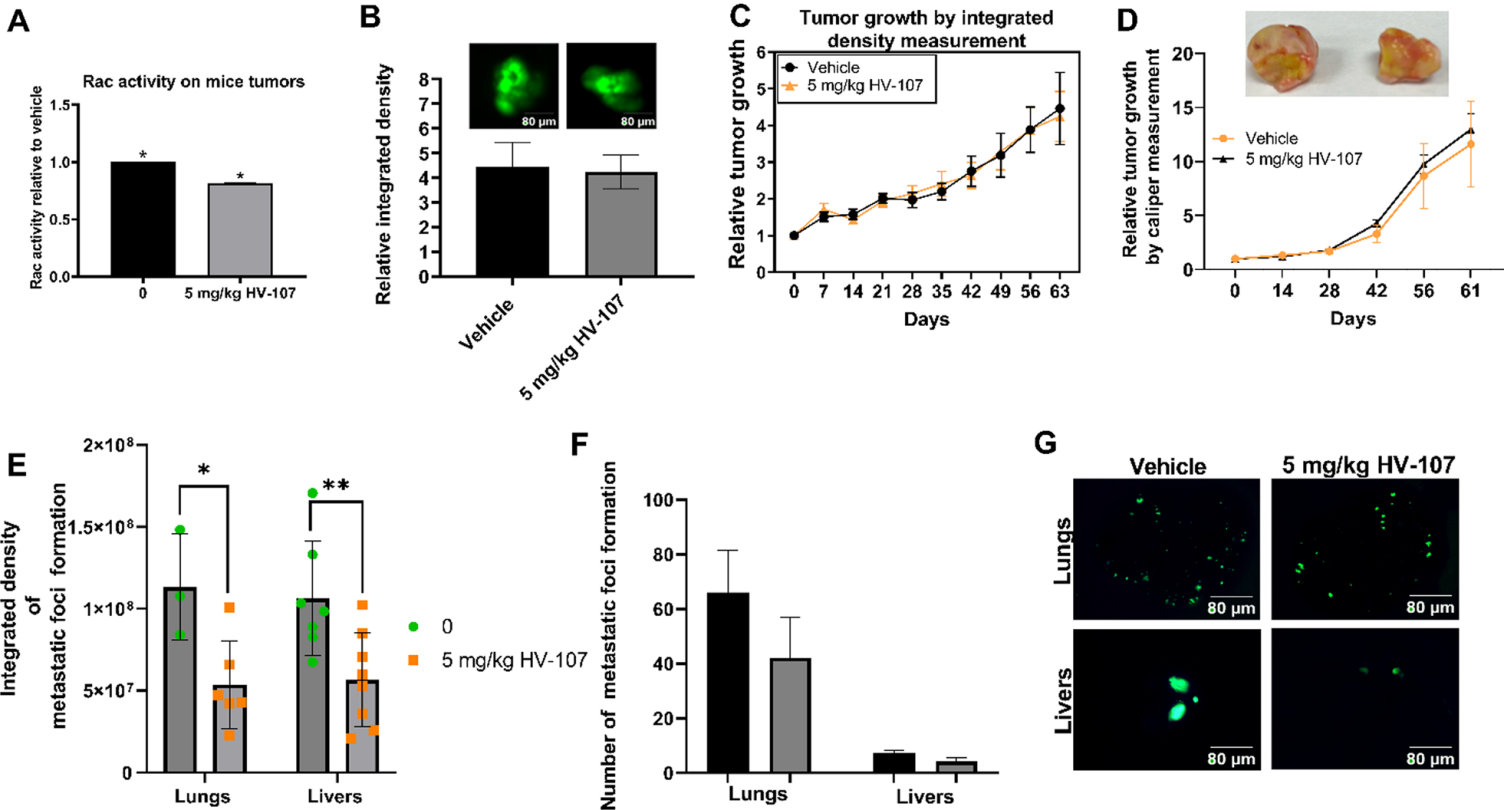
In a xenograft mouse model of breast cancer, the administration of HV-107 results in reduced metastasis formation but does not affect the primary tumor growth. Severe combined immune deficiency (SCID) mice were injected with GFP-MDA-MB-231 cells into the mammary fat pad and subsequently treated with either a vehicle solution (consisting of 12.5% cremophor, 12.5% ethanol, and 75% PBS) or HV-107 at a dosage of 5 mg/kg body weight, administered intraperitoneally five times a week beginning the tumor size reached 100 mm^3^. **A** The Rac activity in the tumors of the mice was analyzed using GLISA analysis. **B** Fluorescent images of the tumors were captured, and the relative integrated density of the tumors was quantified after 64 days of treatment. **C**, **D** Tumor growth measurement by caliper and intensity of fluorescence, analyzed as a function of days. The tumors that were surgically removed after the 64-day treatment course are presented. **E** Analysis of integrated density of metastatic foci formed in lungs and livers. **F** Average number of metastatic foci formed in lungs and livers. **G** Fluorescent images of metastatic foci formed on lungs and livers. Mean ± S.E. and significant differences from vehicle (*) are shown (p < 0.05). The number of mice in each treatment group was as follows: vehicle (N = 13), 5 mg/kg HV-107 (N = 13)

Nude mice with established GFP-expressing MDA-MB-231 tumors were treated intraperitoneally (i.p.) with either a vehicle or HV-107 at a dosage of 5 mg/kg body weight daily. To assess the effect of HV-107 on Rac activity in the tumors, a GLISA approach was employed, revealing a 20% reduction in mice treated with HV-107 (Fig. 7A). Tumor size was measured using a caliper, and the integrated fluorescence density in the tumors was also evaluated (Fig. 7B–D). The results demonstrated that HV-107 had no significant impact on tumor growth (Fig. 7B, D, Additional file 15: Fig. S15).

Moreover, the administration of HV-107 led to a 50% decrease in the formation of liver and lung metastases, as determined by integrated density measurements (Fig. 7E–G). Analysis of the number of metastatic foci formed following HV-107 treatment (Fig. 7F) indicated a tendency towards reducing both the total number of metastatic foci and the size of the formed metastases.

The toxicity of HV-107 treatment was assessed by daily behavioral and physical observations of mice. No abnormal behavior, skin, or eye appearance was observed. The mice’s weight and hepatic enzyme levels (ALT, ALP, and AST), assessed from blood samples collected via cardiac puncture at the end of the study, remained without changes during the study, as presented in Additional file 14: Fig. S14.

Changes in liver weight, correlated with liver damage, were not observed. To summarize, HV-107 treatment was found to decrease metastasis formation in lungs and liver, but did not have significant effects on primary tumor growth.

## Discussion

Currently, various strategies have been devised to inhibit Rac activity. These approaches primarily focus on disrupting the interaction between Rac and its upstream regulators known as GEFs (such as NSC23766, ITX3, AZA197) or inhibiting nucleotide binding to Rac (e.g., EHT-1864, ML141). Typically, these interventions involve using concentrations ranging from 5 to 100 µM (Levay et al. 2013; Melzer et al. 2019). However, the use of these inhibitors at indicated high doses has raised concerns regarding their potential off-target effects. Examples include interference with muscarinic acetylcholine receptors. (Dütting et al. 2015; Levay et al. 2013). In this study, HV-107 demonstrated 50% inhibition of Rac within a range of 250–500 nM, suggesting a potential reduction in off-target effects compared to existing inhibitors. Additionally, HV-107 effective doses were found to be 2 to 4 times lower than the leading compound EHop-016. The IC50 value for Rac inhibition with EHop-016 is 1.1 µM, while an effective concentration of 4 µM is required to decrease migration, which is associated with multiple off-target effects. In contrast, 250–500 nM HV-107 effectively inhibited invasion of breast cancer cells, exhibiting minimal off-target effects on related signalling pathways. Based on these findings, HV-107 appears to be a more viable and safer option for inhibiting metastasis formation compared to the parent compound EHop-016. Furthermore, in vivo studies conducted in mice demonstrated that HV-107 effectively inhibited metastasis formation at a concentration of 5 mg/kg body weight, while the lead compound required a higher dose of 25 mg/kg. The observed variations in response between MDA-MB-231 and MDA-MB-468 cells were expected due to the different expression levels of Rac isoforms (Melzer et al. 2019; Subik et al. 2010; Eiden and Ungefroren 2021). These differences need to be considered in the further development of treatment protocols.

The study identified that HV-107 decreased viability in non-cancerous cells without inducing cell death at concentrations exceeding 1000 nM. However, in TNBC cells, HV-107 reduced viability by 20% within the concentration range of 250–500 nM.

Flow cytometric analysis of the cell cycle demonstrated that HV-107 treatment led to a reduction in the G1/S phase population, accompanied by an increase in the sub-G1 population in both investigated cell lines. The expression of Cyclin D1 and Bcl2 was unaffected by HV-107, except at a concentration of 2000 nM. As MDA-MB-468 cells lack the Retinoblastoma (Rb) protein, while MDA-MB-231 cells express it, differences in apoptotic response between the two cell lines were expected. Consistent with our findings, a previous study using NCS23766 (the first identified Rac inhibitor) revealed, that cells lacking Rb are more likely to undergo apoptosis without significant effects on cyclin D1 and Bcl2 expression (Yoshida et al. 2010). Instead, the downregulation of X-linked inhibitor of apoptosis (XIAP) and survivin, two pro-survival proteins, under Rac inhibition by NCS23766 was shown to promote cell death in triple-negative breast cancer cells regardless of Bcl2 and cyclin D1 expression (Yoshida et al. 2010). Moreover, alternative apoptotic pathways, including the extrinsic pathway mediated by death receptors or the intrinsic pathway regulated by mitochondrial factors, can be activated independently of Bcl2 (Lossi 2022). These pathways involve various pro-apoptotic proteins, such as caspases, cytochrome c, as well as members of the Bcl2 protein family like Bax and Bak, which can initiate apoptosis without direct involvement of Bcl2. In line with our findings, a study by Liang Su et al. demonstrated that the treatment of leukemic cells with resveratrol enhances Cdc42 activation, leading to apoptosis through an increase in Fas ligand (FasL) expression. Therefore, future investigations will focus on analyzing the mechanisms of cell death induced by HV-107(Su et al. 2005).

Previously it was shown that inhibition of Rac1 negatively affects the migration of cancerous cells (Montalvo-Ortiz et al. 2012; Kunschmann et al. 2019; Medina et al. 2022). Thus, we tested the potential of HV-107 to inhibit cell migration and extracellular matrix degradation. In our study, treatment with HV-107 exhibited a notable decrease in migration, invasion, and extracellular matrix degradation (Fig. 4A–I), which corresponded to the inhibition of Rac activity. Although HV-107 did not strongly affect the formation of invadopodia (IF), there was a significant reduction in invadopodia activity (IA) even at low concentrations, leading to a modest inhibition of Rac. This is consistent with other studies indicating that Rac inhibition primarily affects matrix degradation by invadopodia rather than their number (Nuñez et al. 2021).

Furthermore, the study demonstrated that HV-107 also affected Pyk2 and FAK signalling in TNBC cells, which are crucial regulators of migration and extracellular matrix degradation in various cancers (Naser et al. 2018; Chan et al. 2009; Genna et al. 2018; Chang et al. 2007; Tomar and Schlaepfer 2010). Previous studies have highlighted the role of Pyk2 in controlling the secretion and activation of metalloproteinases (MMPs) in invadopodia, while the depletion of FAK results in an active pool of Src, leading to increased phosphorylation of substrates in invadopodia, impaired focal adhesion dynamics, and enhanced formation and dynamics of invadopodia (Tomar and Schlaepfer 2010; Bae et al. 2014). Our findings are consistent with the existing literature, as HV-107 downregulates FAK signaling in MDA-MB-231 and Pyk2 signaling in both cell lines, resulting in decreased invadopodia activity. In the case of MDA-MB-468, we also observed an upregulation of Pyk2 and FAK phosphorylation at HV-107 concentrations exceeding 1000 nM, which influenced invadopodia activity and maturation. This synchronized mechanism is crucial for successful migration and invasion. The literature suggests that Src serves as a common partner between FAK and Pyk2 (Genna et al. 2018). When FAK is active, it recruits Src to focal adhesions, whereas a decrease in FAK activity leads to the release of Src, which Pyk2 recruits as a precursor for invadopodia maturation. Therefore, the deregulation of both FAK and Pyk2 disrupts this mechanism of action, ultimately impacting migration and invasion processes.

Concentrations of HV-107, exceeding 1000 nM, caused the over-activation of Rho and Cdc42, similar to the findings from a previous study using Ehop-016, where Rho over-activation was observed (Montalvo-Ortiz et al. 2012). Rac, Rho, and Cdc42 operate in a highly coordinated manner, forming a synchronized machinery. Disruption of this machinery leads to the dysregulation of cytoskeletal structures such as filopodia, stress fibers, and lamellipodia formation, thereby affecting cell migration and invasion. The upregulation of Rho in response to Rac inhibition is consistent with the notion that Rac activation is required to regulate Rho activity to facilitate the forward movement of the cell (Parri and Chiarugi 2010; Hanna and El-Sibai 2013). In this study, an increase in Cdc42 levels at the highest concentrations of HV-107, exceeding 1000 nM, was observed. This increase is likely related to the activation of FAK and PAK signalling, and a positive feedback loop between them. FAK activation has been linked to the activation of WNT signalling, which, in turn, has been shown to activate Cdc42 GTPase (Mezzacappa et al. 2012; Wörthmüller and Rüegg 2020). Therefore, the observed effect on Cdc42 activation at 1000 nM HV-107 may be associated with FAK activation. Alternatively, an increase in the activation of GEFs (guanine nucleotide exchange factors) could also contribute to the upregulation of Cdc42.

The effect of HV-107 on the activity of PAK, a downstream effector of both Rac and Cdc42 (Best et al. 2022), was also investigated. The investigation demonstrated that treatment with HV-107 at concentrations exceeding 1000 nM led to an increase in PAK1/2 and Cdc42 signalling in both the MDA-MB-231 and MDA-MB-468 cell lines. Furthermore, Alok et al. found that PAK protein can act as a linker for the activation of FAK through the activation of Src-3 and 4, and a positive feedback loop between FAK and PAK exists to sustain FAK activation (Tomar and Schlaepfer 2010; Bae et al. 2014). PAK activation has also been shown to promote cytostatic effects on cancerous cells (Roig et al. 2001).

The in vivo experiments revealed that HV-107 suppressed Rac activity in mouse tumors by approximately 20%. Furthermore, HV-107 did not cause a significant decrease in tumor size but reduce lung and liver metastasis growth by 50%, (Fig. 7B–G). These findings support the notion that HV-107 may act as a cytostatic drug, inhibiting cell proliferation and metastasis without inducing primary tumor shrinkage. Toxicity analysis of HV-107 showed no significant toxicity toward mice at the tested concentrations.

Our research findings indicate that HV-107 holds promise as a therapeutic drug for the treatment of metastatic triple-negative breast cancer (TNBC). Through our study, we have established the optimal working concentrations of HV-107, which fall within the range of 250–500 nM for in vitro studies and 5 mg/kg for in vivo studies. At these concentrations, we observed a significant down-regulation of Rac activity while minimizing off-target effects. Nevertheless, it is worth exploring the potential efficacy of HV-107 in treating other cancers, such as HER2 + breast cancer, where the overexpression or hyperactivation of Rac has been associated with acquired resistance to chemotherapy (Sun et al. 2021). To advance the development of HV-107 as a therapeutic agent, future studies should prioritize the assessment of off-target signaling pathways. Furthermore, evaluating HV-107 on patient-derived cell lines would offer a more clinically relevant model for assessing both its efficacy and safety and could provide valuable information regarding the potential use of these drugs in personalized medicine approaches for cancer therapy.

## Conclusions

In conclusion, study showed that HV-107 exhibited significant inhibitory effects on Rac activity in MDA-MB-231 and MDA-MB-468 cells in vitro at concentrations ranging from 250 to 2000 nM. Treatment with HV-107 at concentrations of 100 nM and higher resulted in the inhibition of migration, invasion, and invadopodia activity. Furthermore, HV-107 at concentrations of 500 nM and higher demonstrated a dose-dependent reduction in cell viability and induced cell death specifically in TNBC cells, while non-cancerous cells showed this effect only at concentrations exceeding 1000 nM. HV-107 at concentrations exceeding 1000 nM upregulated the signaling pathways of PAK1, PAK2, FAK, Pyk2, Cdc42, and Rho, while Pyk2 was downregulated in the concentration range of 100–500 nM. Through our investigations, we determined that the optimal concentrations for HV-107 ranged from 250 to 500 nM in vitro, as they effectively inhibited Rac activity with minimal off-target effects on signaling pathways associated with Rho-GTPases.In our in vivo studies, administration of 5 mg/kg HV-107 demonstrated a 20% reduction in Rac activity within tumors and a significant decrease (50%) in metastasis formation in the lungs and liver, without any observed toxicity towards the mice at the tested doses.

## Supplementary Information

**Additional file 1: Fig. S1.** Uncropped western blot membranes and loading controls for identification of Rac-GTP and Rac total expression on MDA-MB-231 and MDA-MB-468 cells treated with HV-107, in support of Fig. 1 A-H. Actin was used as loading control.

**Additional file 2: Fig. S2.** Uncropped western blot membranes and loading controls for identification of Vav2 total expression on MDA-MB-231 cells treated with HV-107, in support of Fig. 1I–J. Actin was used as loading control.

**Additional file 3: Fig. S3.** Uncropped western blot membranes for detection of cyclin D1 and Bcl2 in MDA-MB-231 and MDA-MB-468 cells treated with HV-107 at concentrations ranging from 0 to 2000 nM, in support of Fig. 3. Actin was used as a loading control.

**Additional file 4: Fig. S4**. Representative fluorescent images for migration assays of HV-107-treated MDA-MB-231 and MDA-MB-468 cells, in support of Fig. 4A-B. Scale bar 80 µm.

**Additional file 5: Fig. S5.** Representative fluorescent images for invadopodia formation and invasion assays for MDA-MB-231 and MDA-MB-468 cells treated with HV-107 ranging from 0 to 2000 nM, in support of Fig. 4C-I. Green color: FITC gelatin with black spot as degradation sites. Red color: actin structures stained with TRITC-conjugated phalloidin. Blue color: nuclear staining with DAPI. Scale bar 80μm.  

**Additional file 6: Fig. S6.** Uncropped western blot membranes for total Cdc42 in MDA-MB-231 and MDA-MB-468 cells treated with HV-107 ranging from 0 to 2000 nM, in support of Fig. 5 Actin was used as a loading control.

**Additional file 7: Fig. S7**. Uncropped westen blot membranes for total Rho in MDA-MB-231 and MDA-MB-468 cells treated with HV-107 ranging from 0-2000 nM, in support of Fig.  5. Actin was used as a loading control.

**Additional file 8: Fig. S8**. Confocal images of stress fibers and analysis of filopodia length and number in MDA-MB-231 and MDA-MB-468 cells upon treatment with HV-107, in support of Fig. 5. Scale bar 50 µm.

**Additional file 9: Fig. S9.** Uncropped western blot membranes for total and phosphorylated PAK1 and PAK2 in MDA-MB-231 cells, treated with HV-107, ranging from 0 to 2000 nM, in support of Fig. 5. Actin was used as a loading control.

**Additional file 10: Fig. S10.** Uncropped western blot membranes for total and phosphorylated PAK1 and PAK2 in MDA-MB-468 cells, treated with HV-107, ranging from 0 to 2000 nM, in support of Fig. 5. Actin was used as a loading control.

**Additional file 10: Fig. S11.**. Uncropped western blot membranes for total PAK2 in MDA-MB-468 cells, treated with HV-107,ranging from 0-2000 nM, in support of Fig. 5. Actin was used as a loading control

**Additional file 12: Fig. S12.** Uncropped western blot membranes for total and phosphorylated FAK in MDA-MB-231 cells, treated with HV-107, ranging from 0 to 2000 nM, in support of Fig. 6. Actin was used as a loading control.

**Additional file 13: Fig. S13.** Uncropped western blot membranes for total and phosphorylated FAK in MDA-MB-468 cells, treated with HV-107, ranging from 0 to 2000 nM, in support of Fig. 6. Actin was used as a loading control.

**Additional file 14: Fig. S14.** Uncropped western blot membranes for total and phosphorylated Pyk2 in MDA-MB-231 and MDA-MB-468 cells, treated with HV-107, ranging from 0 to 2000 nM, in support of Fig. 6. Actin was used as a loading control.

**Additional file 15: Fig. S15.** Assessment of HV-107 toxicity in xenograft breast cancer model. Tumor weight, liver weight, hepatic enzymes levels, and mice body weightare presented for mice treated with vehicle or 5 mg/kg body weight HV-107, in support of Fig. 8.

## Data Availability

The data generated in this study are available within the article and its supplementary data files.

## Abbreviations

ALP: Aspartate aminotransferase
ALT: Alanine transaminase
AST: Aspartate aminotransferase
Bcl2: B-cell lymphoma 2
Cdc42: Cell division control protein 42
EGF: Epidermal growth factor
ERþ: Estrogen receptor–positive
EMT: Epithelial to mesenchymal transition
FAK: Focal adhesion kinase
GEF: Guanine nucleotide exchange factor
GFP: Green fluorescent protein
HGF: Hepatocyte growth factor
IA: Invadopodia activity
IF: Invadopodia formation
PAK: P-21 activated kinase
PDGF: Platelet derived growth factor
Pyk2: Proline-rich tyrosine kinase
Rac: Ras-related C3 botulium toxin substrate
SCID: Severe combined immunodeficient mice
TNBC: Triple negative breast cancer
WHO: World health organization
XIAP: X-linked inhibitor of apoptosis protein
