# Supplementary figures and images for: The Rac inhibitor HV-107 as a potential therapeutic for metastatic breast cancer
Source: Mol Med. 2023 Jun 14;29:75. doi: 10.1186/s10020-023-00678-7 (PMC10268403; doi:10.1186/s10020-023-00678-7)

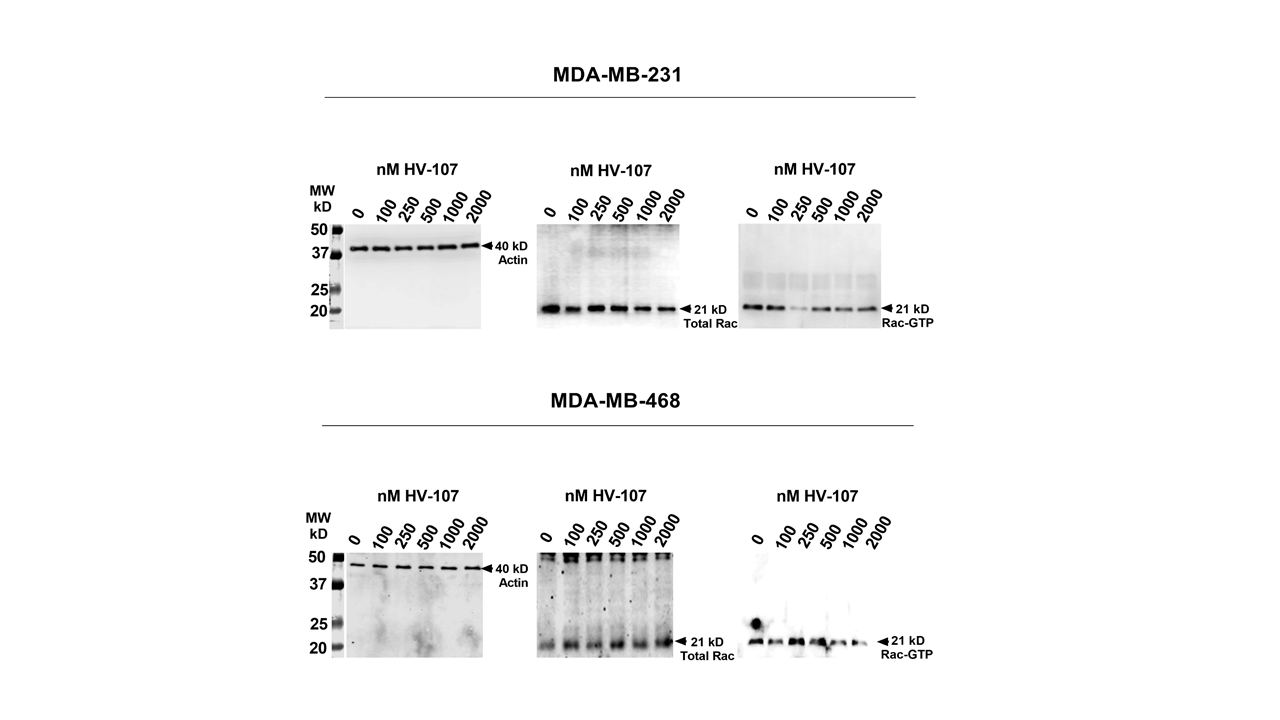

Supplement: Supplementary file 1 — Additional file 1: Fig. S1. Uncropped western blot membranes and loading controls for identification of Rac-GTP and Rac total expression on MDA-MB-231 and MDA-MB-468 cells treated with HV-107, in support of Fig. 1 A-H. Actin was used as loading control. [file 10020_2023_678_MOESM1_ESM.tif]

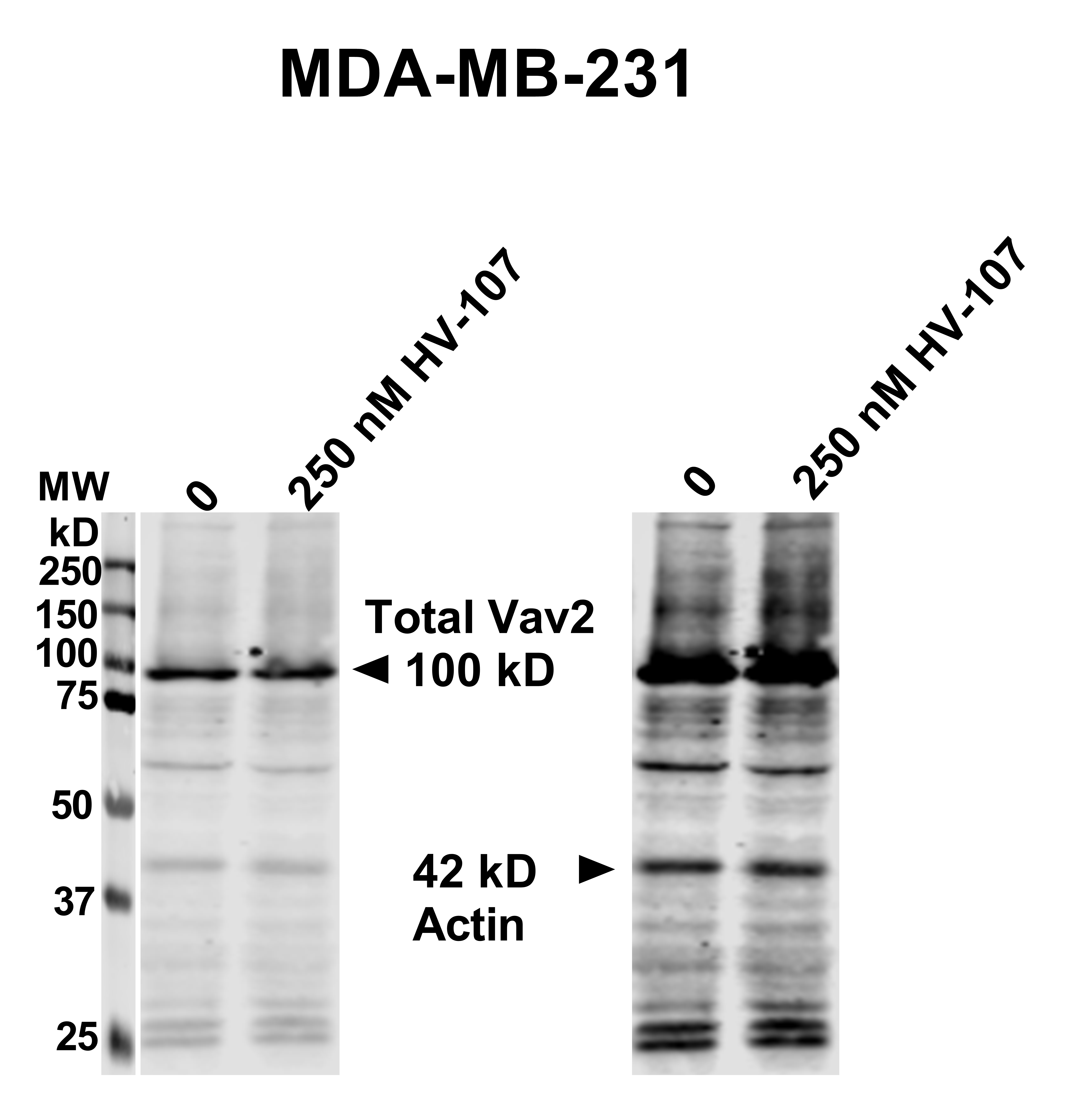

Supplement: Supplementary file 2 — Additional file 2: Fig. S2. Uncropped western blot membranes and loading controls for identification of Vav2 total expression on MDA-MB-231 cells treated with HV-107, in support of Fig. 1I–J. Actin was used as loading control. [file 10020_2023_678_MOESM2_ESM.tif]

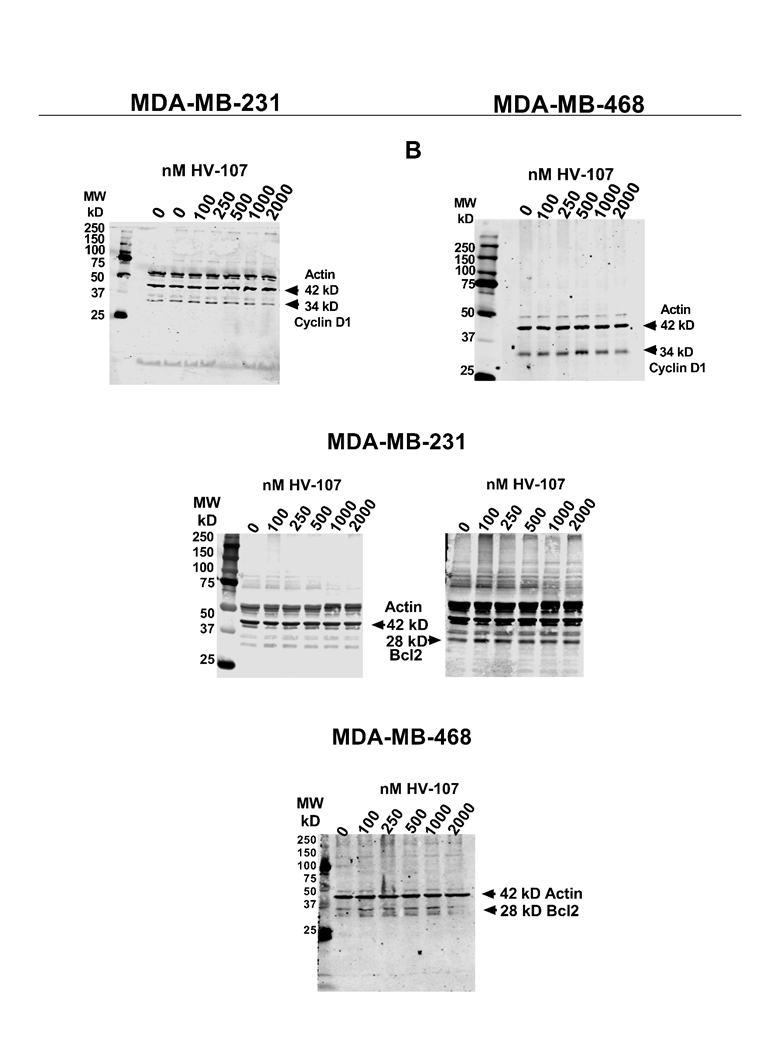

Supplement: Supplementary file 3 — Additional file 3: Fig. S3. Uncropped western blot membranes for detection of cyclin D1 and Bcl2 in MDA-MB-231 and MDA-MB-468 cells treated with HV-107 at concentrations ranging from 0 to 2000 nM, in support of Fig. 3. Actin was used as a loading control. [file 10020_2023_678_MOESM3_ESM.tif]

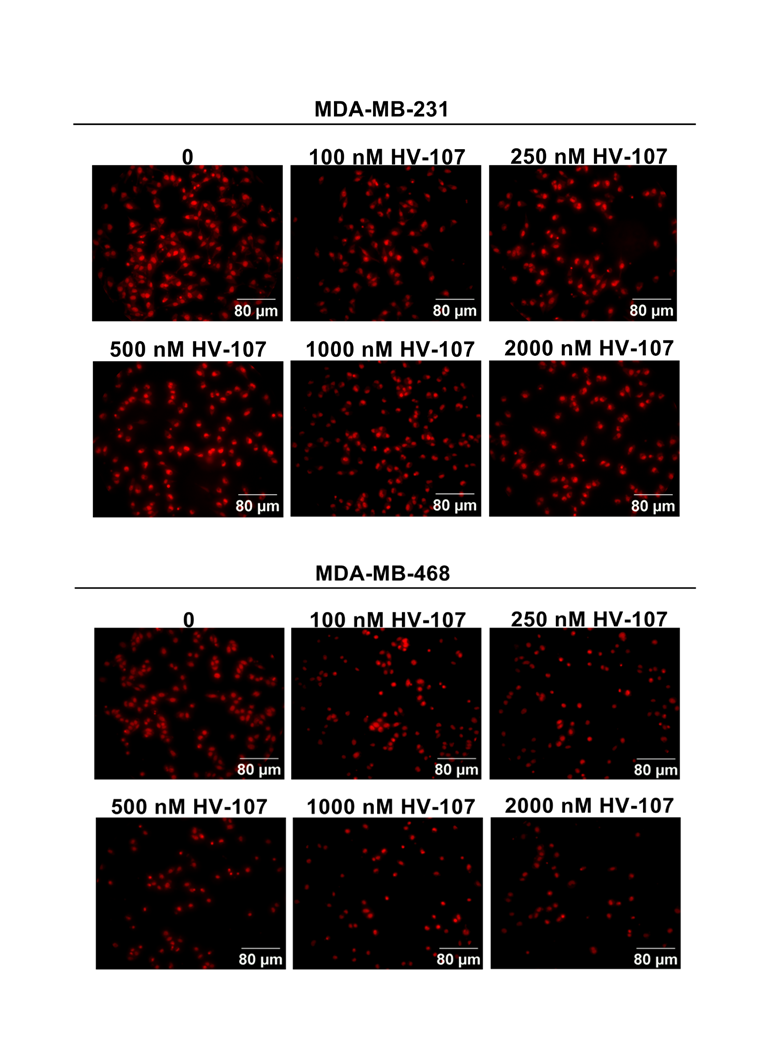

Supplement: Supplementary file 4 — Additional file 4: Fig. S4. Representative fluorescent images for migration assays of HV-107-treated MDA-MB-231 and MDA-MB-468 cells, in support of Fig. 4A-B. Scale bar 80 µm. [file 10020_2023_678_MOESM4_ESM.tif]

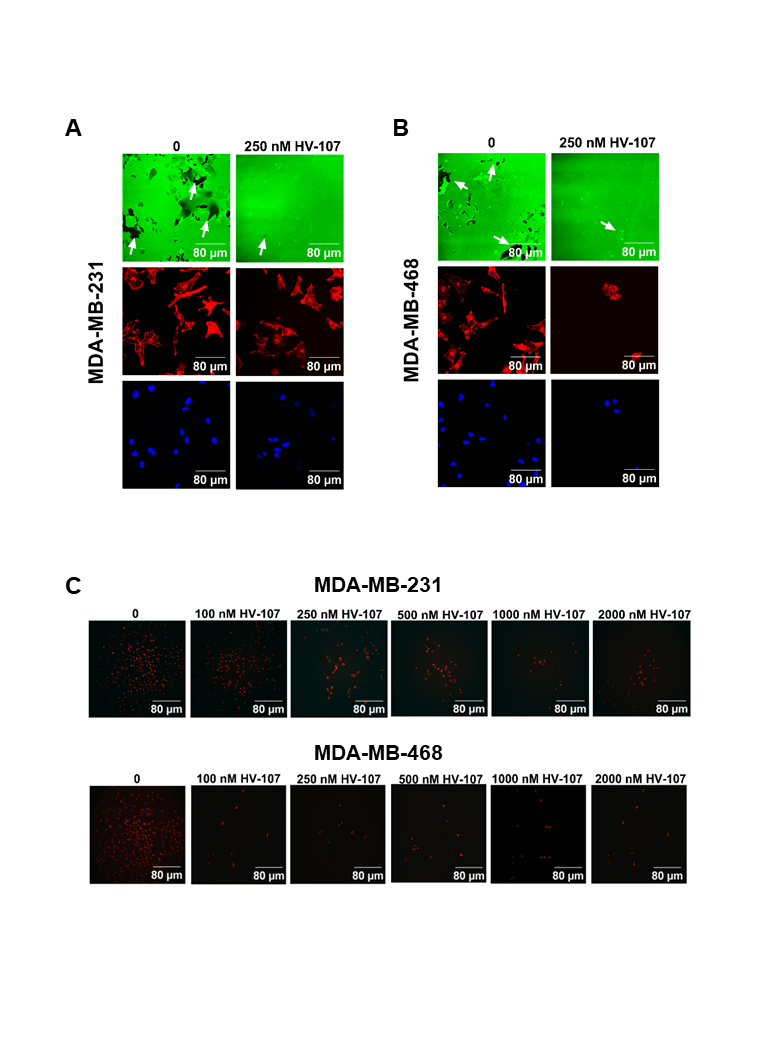

Supplement: Supplementary file 5 — Additional file 5: Fig. S5. Representative fluorescent images for invadopodia formation and invasion assays for MDA-MB-231 and MDA-MB-468 cells treated with HV-107 ranging from 0 to 2000 nM, in support of Fig. 4C-I. Green color: FITC gelatin with black spot as degradation sites. Red color: actin structures stained with TRITC-conjugated phalloidin. Blue color: nuclear staining with DAPI. Scale bar 80μm. [file 10020_2023_678_MOESM5_ESM.tif]

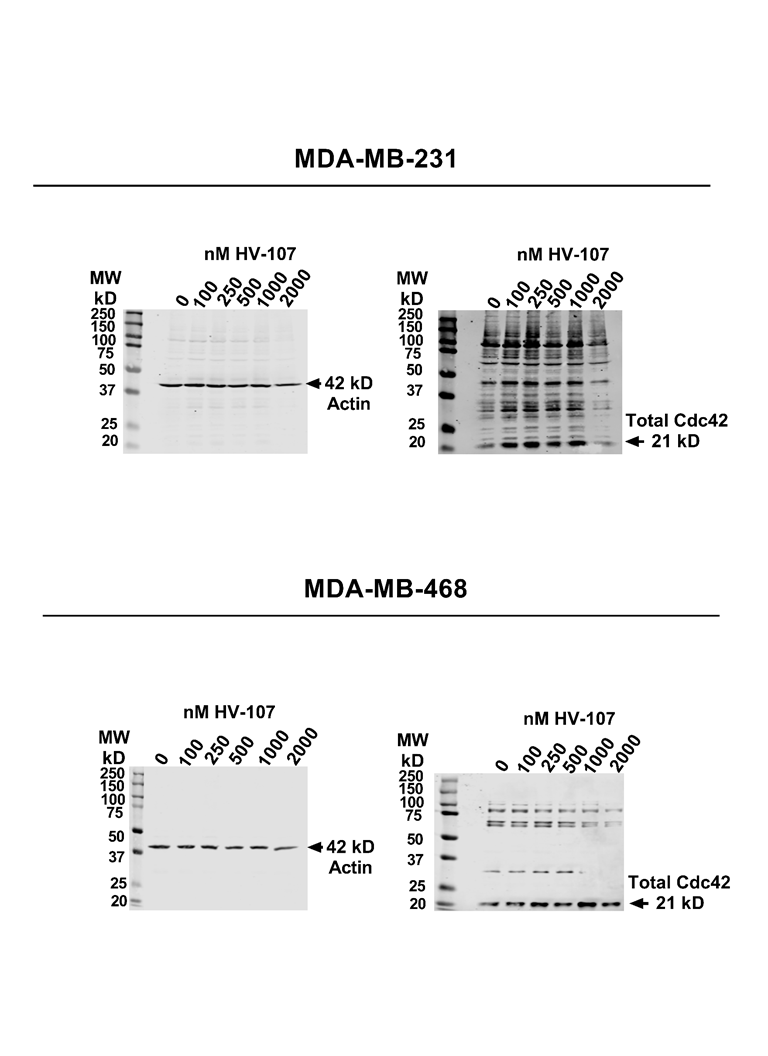

Supplement: Supplementary file 6 — Additional file 6: Fig. S6. Uncropped western blot membranes for total Cdc42 in MDA-MB-231 and MDA-MB-468 cells treated with HV-107 ranging from 0 to 2000 nM, in support of Fig. 5 Actin was used as a loading control. [file 10020_2023_678_MOESM6_ESM.tif]

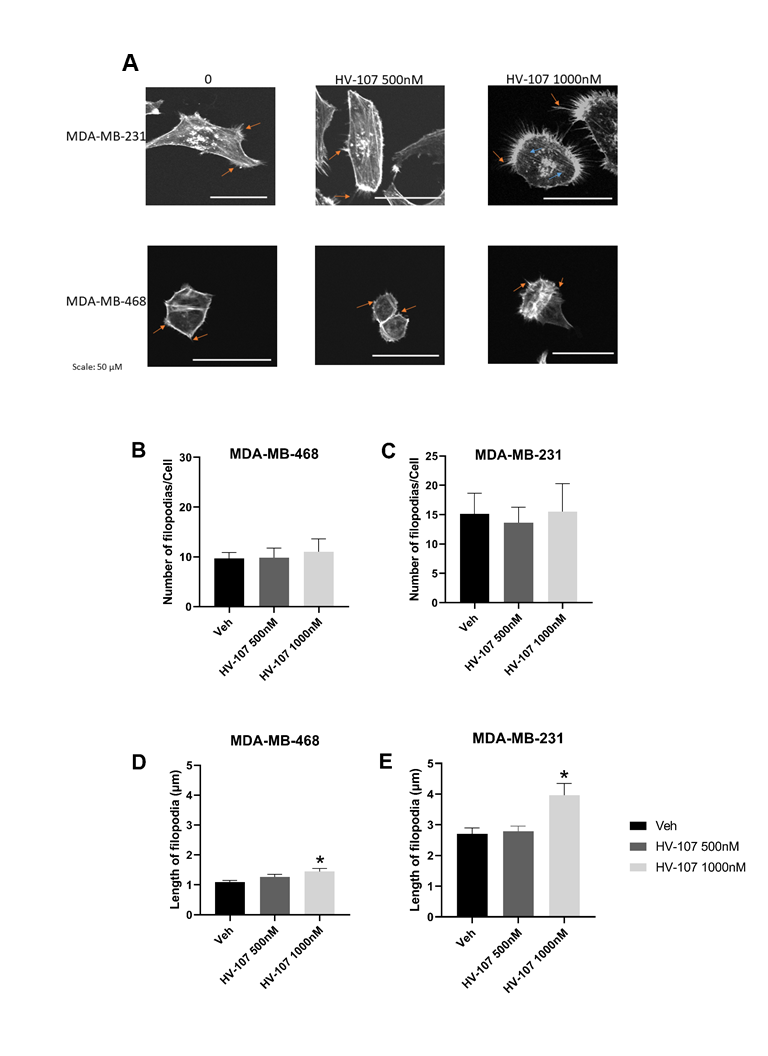

Supplement: Supplementary file 8 — Additional file 8: Fig. S8. Confocal images of stress fibers and analysis of filopodia length and number in MDA-MB-231 and MDA-MB-468 cells upon treatment with HV-107, in support of Fig. 5. Scale bar 50 µm. [file 10020_2023_678_MOESM8_ESM.tif]

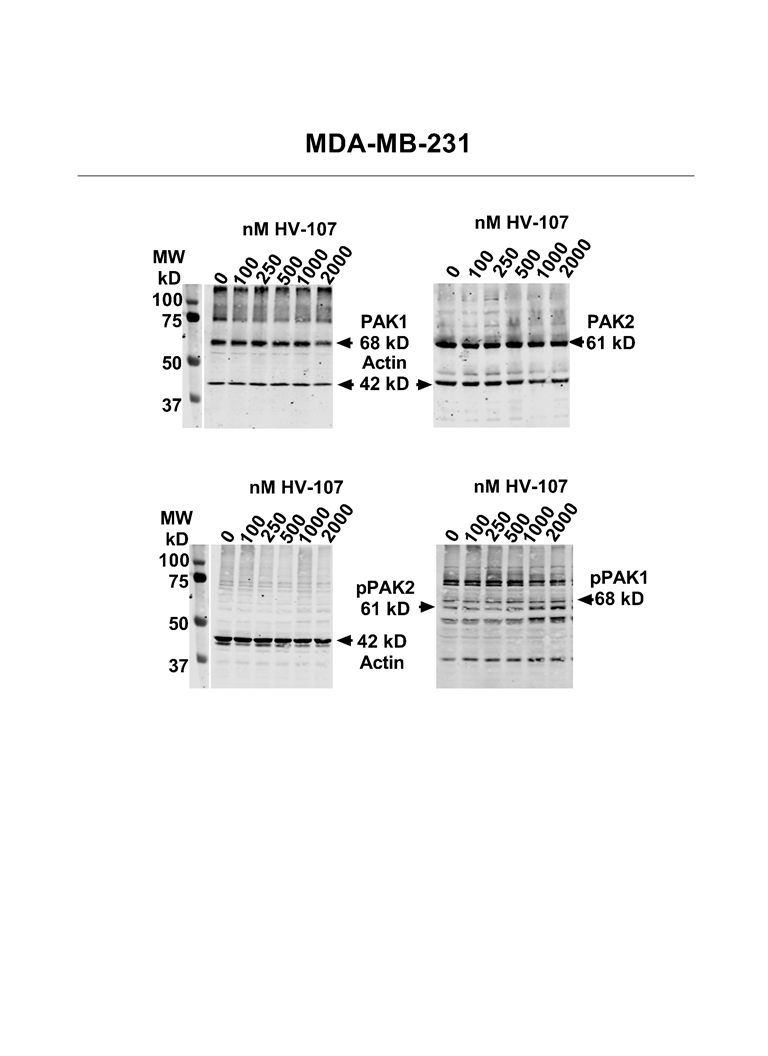

Supplement: Supplementary file 9 — Additional file 9: Fig. S9. Uncropped western blot membranes for total and phosphorylated PAK1 and PAK2 in MDA-MB-231 cells, treated with HV-107, ranging from 0 to 2000 nM, in support of Fig. 5. Actin was used as a loading control. [file 10020_2023_678_MOESM9_ESM.tif]

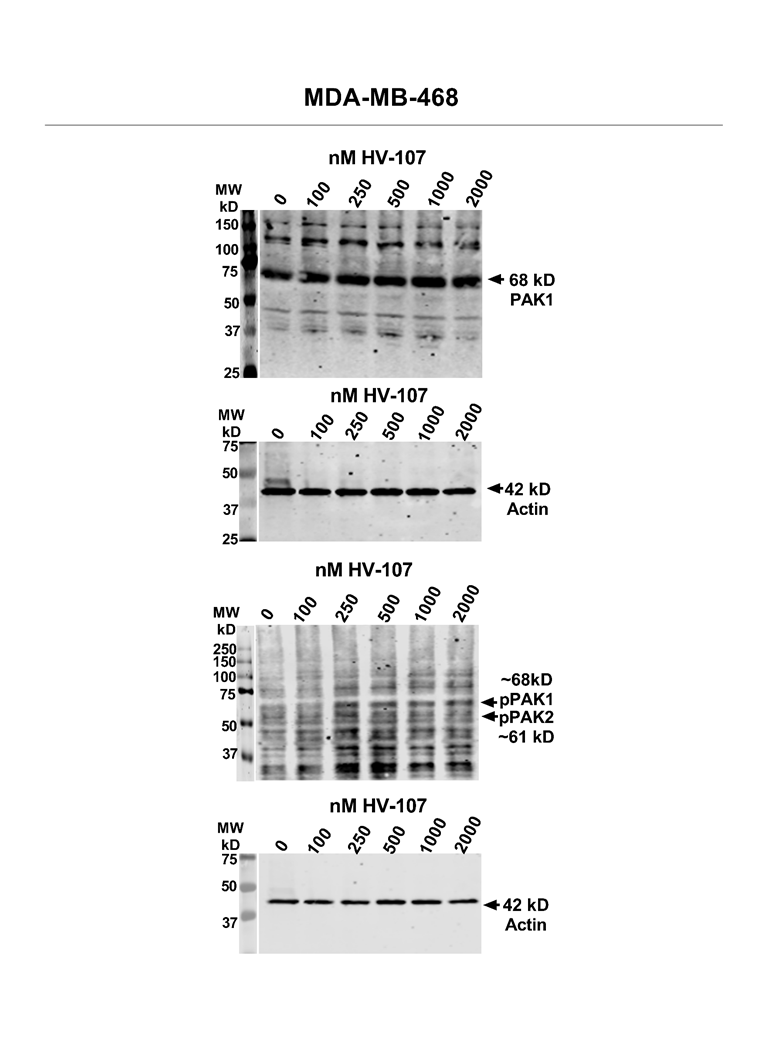

Supplement: Supplementary file 10 — Additional file 10: Fig. S10. Uncropped western blot membranes for total and phosphorylated PAK1 and PAK2 in MDA-MB-468 cells, treated with HV-107, ranging from 0 to 2000 nM, in support of Fig. 5. Actin was used as a loading control. [file 10020_2023_678_MOESM10_ESM.tif]

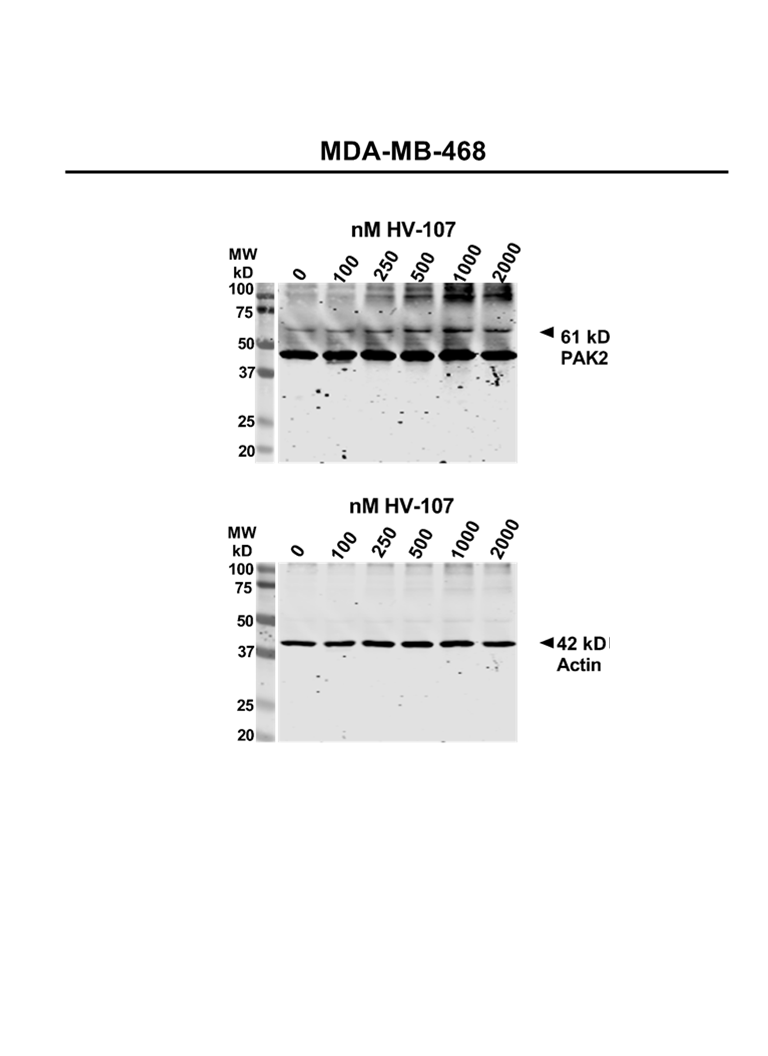

Supplement: Supplementary file 11 — Additional file 10: Fig. S11.. Uncropped western blot membranes for total PAK2 in MDA-MB-468 cells, treated with HV-107,ranging from 0-2000 nM, in support of Fig. 5. Actin was used as a loading control [file 10020_2023_678_MOESM11_ESM.tif]

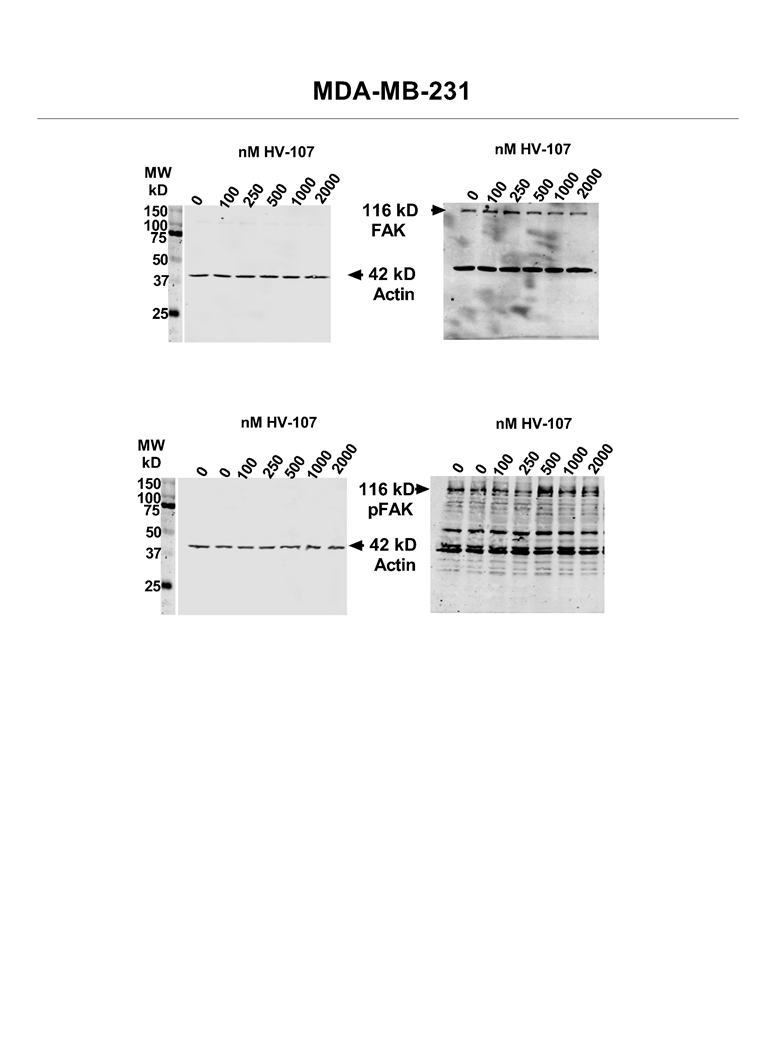

Supplement: Supplementary file 12 — Additional file 12: Fig. S12. Uncropped western blot membranes for total and phosphorylated FAK in MDA-MB-231 cells, treated with HV-107, ranging from 0 to 2000 nM, in support of Fig. 6. Actin was used as a loading control. [file 10020_2023_678_MOESM12_ESM.tif]

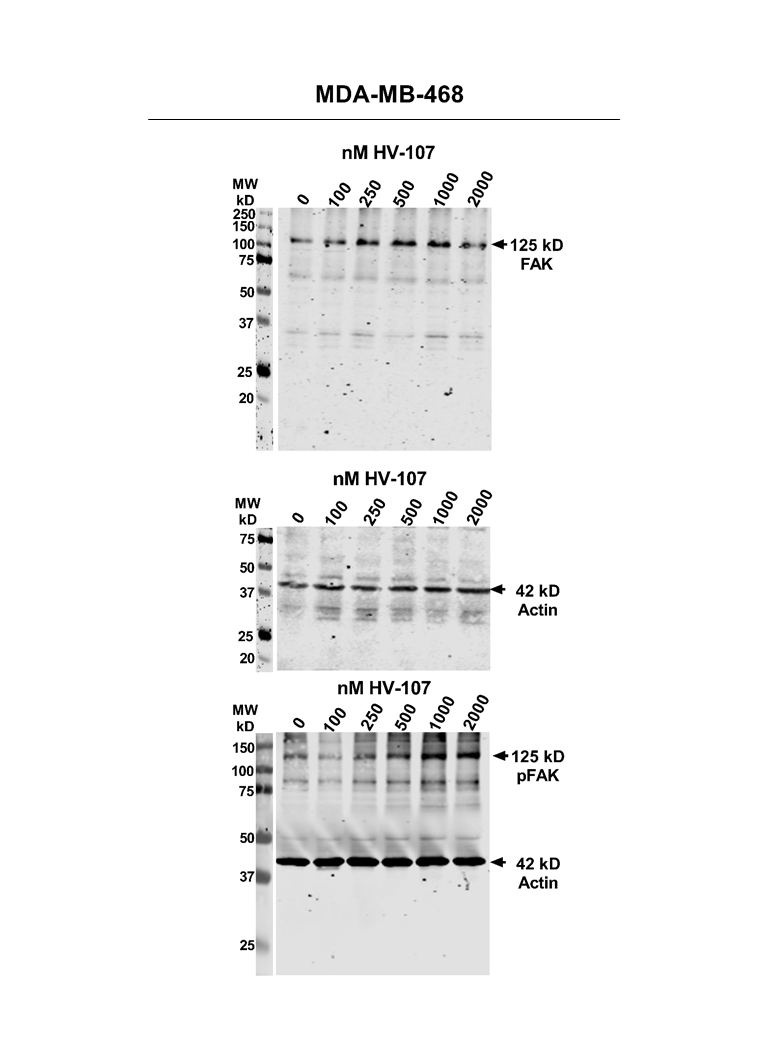

Supplement: Supplementary file 13 — Additional file 13: Fig. S13. Uncropped western blot membranes for total and phosphorylated FAK in MDA-MB-468 cells, treated with HV-107, ranging from 0 to 2000 nM, in support of Fig. 6. Actin was used as a loading control. [file 10020_2023_678_MOESM13_ESM.tif]

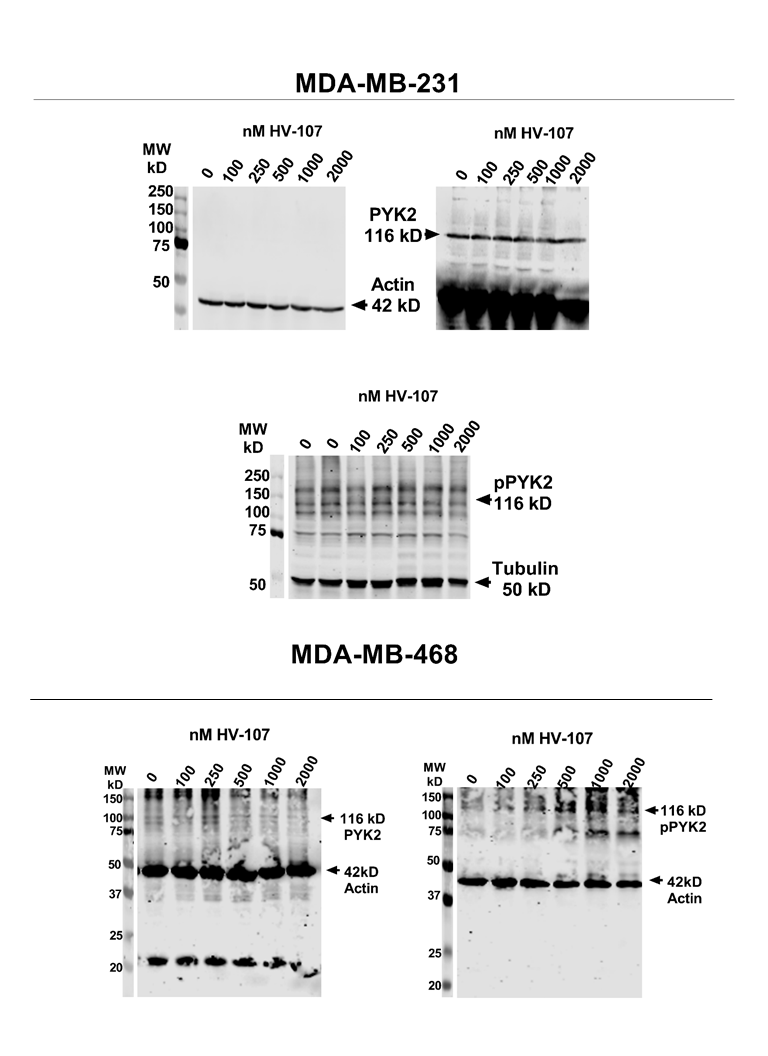

Supplement: Supplementary file 14 — Additional file 14: Fig. S14. Uncropped western blot membranes for total and phosphorylated Pyk2 in MDA-MB-231 and MDA-MB-468 cells, treated with HV-107, ranging from 0 to 2000 nM, in support of Fig. 6. Actin was used as a loading control. [file 10020_2023_678_MOESM14_ESM.tif]

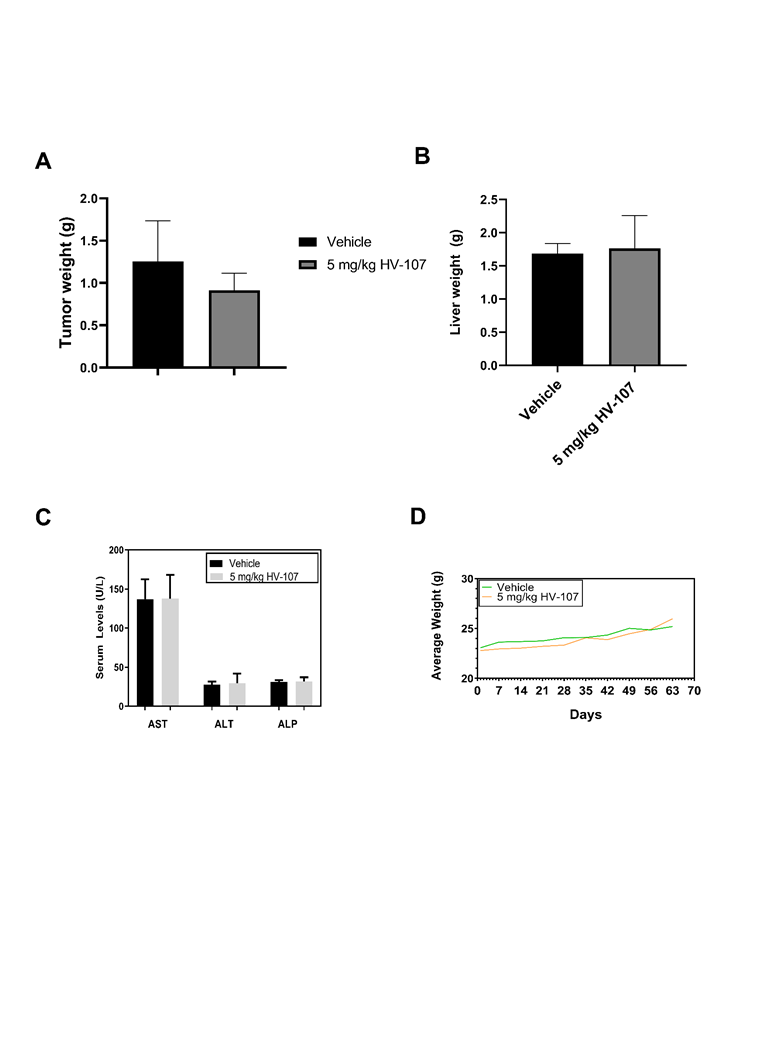

Supplement: Supplementary file 15 — Additional file 15: Fig. S15. Assessment of HV-107 toxicity in xenograft breast cancer model. Tumor weight, liver weight, hepatic enzymes levels, and mice body weightare presented for mice treated with vehicle or 5 mg/kg body weight HV-107, in support of Fig. 8. [file 10020_2023_678_MOESM15_ESM.tif]
